# Intragraft memory-like CD127^hi^CD4^+^Foxp3^+^ Tregs maintain transplant tolerance

**DOI:** 10.1172/jci.insight.169119

**Published:** 2024-03-22

**Authors:** Yuanfei Zhao, Leigh Nicholson, Hannah Wang, Yi Wen Qian, Wayne J. Hawthorne, Elvira Jimenez-Vera, Brian S. Gloss, Joey Lai, Adwin Thomas, Yi Vee Chew, Heather Burns, Geoff Y. Zhang, Yuan Min Wang, Natasha M. Rogers, Guoping Zheng, Shounan Yi, Stephen I. Alexander, Philip J. O’Connell, Min Hu

**Affiliations:** 1 Centre for Transplant and Renal Research and; 2 Scientific Platforms, The Westmead Institute for Medical Research, Westmead, New South Wales, Australia.; 3 Centre for Kidney Research, The Children’s Hospital at Westmead, Westmead, New South Wales, Australia.; 4 Renal and Transplant Medicine Unit, Westmead Hospital, Westmead, New South Wales, Australia.; 5 Faculty of Medicine and Health, The University of Sydney, Camperdown, New South Wales, Australia.

**Keywords:** Transplantation, Tolerance

## Abstract

CD4^+^Foxp3^+^ regulatory T cells (Tregs) play an essential role in suppressing transplant rejection, but their role within the graft and heterogeneity in tolerance are poorly understood. Here, we compared phenotypic and transcriptomic characteristics of Treg populations within lymphoid organs and grafts in an islet xenotransplant model of tolerance. We showed Tregs were essential for tolerance induction and maintenance. Tregs demonstrated heterogeneity within the graft and lymphoid organs of tolerant mice. A subpopulation of CD127^hi^ Tregs with memory features were found in lymphoid organs, presented in high proportions within long-surviving islet grafts, and had a transcriptomic and phenotypic profile similar to tissue Tregs. Importantly, these memory-like CD127^hi^ Tregs were better able to prevent rejection by effector T cells, after adoptive transfer into secondary Rag^–/–^ hosts, than naive Tregs or unselected Tregs from tolerant mice. Administration of IL-7 to the CD127^hi^ Treg subset was associated with a strong activation of phosphorylation of STAT5. We proposed that memory-like CD127^hi^ Tregs developed within the draining lymph node and underwent further genetic reprogramming within the graft toward a phenotype that had shared characteristics with other tissue or tumor Tregs. These findings suggested that engineering Tregs with these characteristics either in vivo or for adoptive transfer could enhance transplant tolerance.

## Abstract 2

A subpopulation of memory-like CD127^hi ^Tregs develops in lymphoid-organs and are further reprogrammed within grafts toward a tissue Treg-like phenotype and are critical for maintaining tolerance.

## Graphical abstract

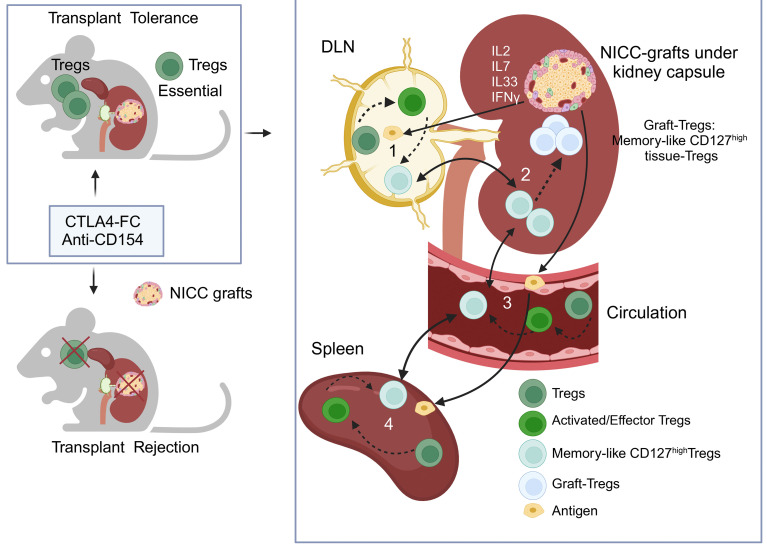

## Introduction

CD4^+^Foxp3^+^ regulatory T cells (Tregs), which play a critical role in controlling immune activation and preventing autoimmune disease (1, 2), are responsible for the maintenance of transplant tolerance (3, 4). The concept of Tregs suppressing the immune response was clarified by Sakaguchi et al., who identified a subset of thymically derived CD4^+^ T cells expressing high levels of IL-2 receptor-α (CD25) that were capable of controlling autoimmunity (1). This was followed by identification of Foxp3 as the key transcription factor for Treg development in the thymus (2). More recently Tregs have been identified outside of the hematological and lymphoid compartments. These tissue-resident Tregs (tissue Tregs) share many of the features of conventional Tregs. In particular, tissue Tregs in visceral adipose tissue (5, 6), intestine (7), and skin (7, 8) have been studied in detail. Apart from their antiinflammatory properties, tissue Tregs have been implicated in normal wound healing, tissue repair, and maintenance of insulin sensitivity. As they reside in multiple tissue compartments, tissue Tregs express tissue-specific phenotypes and specific gene expression profiles. This includes PPARγ expression and regulation of insulin resistance in visceral adipose tissue Tregs (5) or expression of Notch ligand Jagged 1 (JAG1) in skin tissue Tregs (8). Therefore, while all Tregs share common suppressive, antiinflammatory functions, they differ, with tissue-specific functions and unique gene expression profiles that distinguish them from other Treg populations in lymphoid organs and peripheral blood (9).

The adaptive immune system is characterized by its antigen specificity, tight regulation, and immunological memory. Immune memory has been well described in effector T cells and has been identified in tissue Tregs. For example, Tregs with enhanced CD127 expression, as compared with the classic downregulation of CD127 found in naive Tregs, have been identified in a mouse model of skin inflammation where the response to self-antigen imprints regulatory memory in skin tissues (10, 11). In the transplant setting, there are a number of immunomodulatory approaches that induce tolerance to an organ allograft, which are reliant on Tregs that have been shown to be donor antigen specific (4). In experimental settings, this antigen-specific nonresponsiveness has been shown to be powerful and robust and can operate across MHC mismatches (4, 12). In the clinical setting, the development of Treg-dependent tolerance is more challenging. Most clinical trials, such as the ONE Study, have shown Tregs can be administered safely. However, evidence of sufficient potency to allow immunosuppression withdrawal has been lacking (13), but a subgroup of the ONE Study using “antigen-reactive Tregs” did support rejection-free 6-year survival on tacrolimus monotherapy in 3 patients (14). To exploit this strategy clinically, a better understanding of factors that regulate and maintain antigen activated Tregs is required. An important aspect of this is understanding the transcriptomes and phenotypes of Tregs that reside in the graft and lymphoid tissue of transplant-tolerant recipients as well as a better understanding of the environmental factors that drive their differentiation in different anatomical settings.

Here, we used a murine model of porcine neonatal islet cell cluster (NICC) xenotransplant tolerance induced by short-term costimulation blockade. The B7-CD28/cytotoxic T lymphocyte antigen 4 (CTLA4) and CD40-CD154 pathways have been shown to be critically important for T cell activation in transplant rejection. Blocking these 2 pathways by CTLA4-Fc and the anti-CD154 mAb, MHC class I–related protein 1 (CTLA4-Fc/MR1), has been shown to induce graft acceptance in several model systems (4, 15–17). However, the role and characteristics of Tregs with this approach are not fully understood (4, 16). The advantage of this xenotransplant-tolerant model is a high proportion of indirectly activated T cells leading to Treg selection (17, 18). In this model, we have demonstrated previously that long-term tolerance is dependent on the development of activated effector Tregs, and antigen-experienced Tregs are produced in great enough numbers for phenotypic, functional, and genomic analysis (17). In the present study, we showed that Tregs are crucial to transplant tolerance induced by costimulation blockade and demonstrate that memory-like CD127^hi^ tissue Tregs within grafts maintain transplant tolerance. This suggests the possibility of engineering such cells either in vivo or for adoptive transfer to induce and maintain transplant tolerance.

## Results

### Tregs are essential for the induction of porcine NICC graft tolerance.

**Figure 1:**
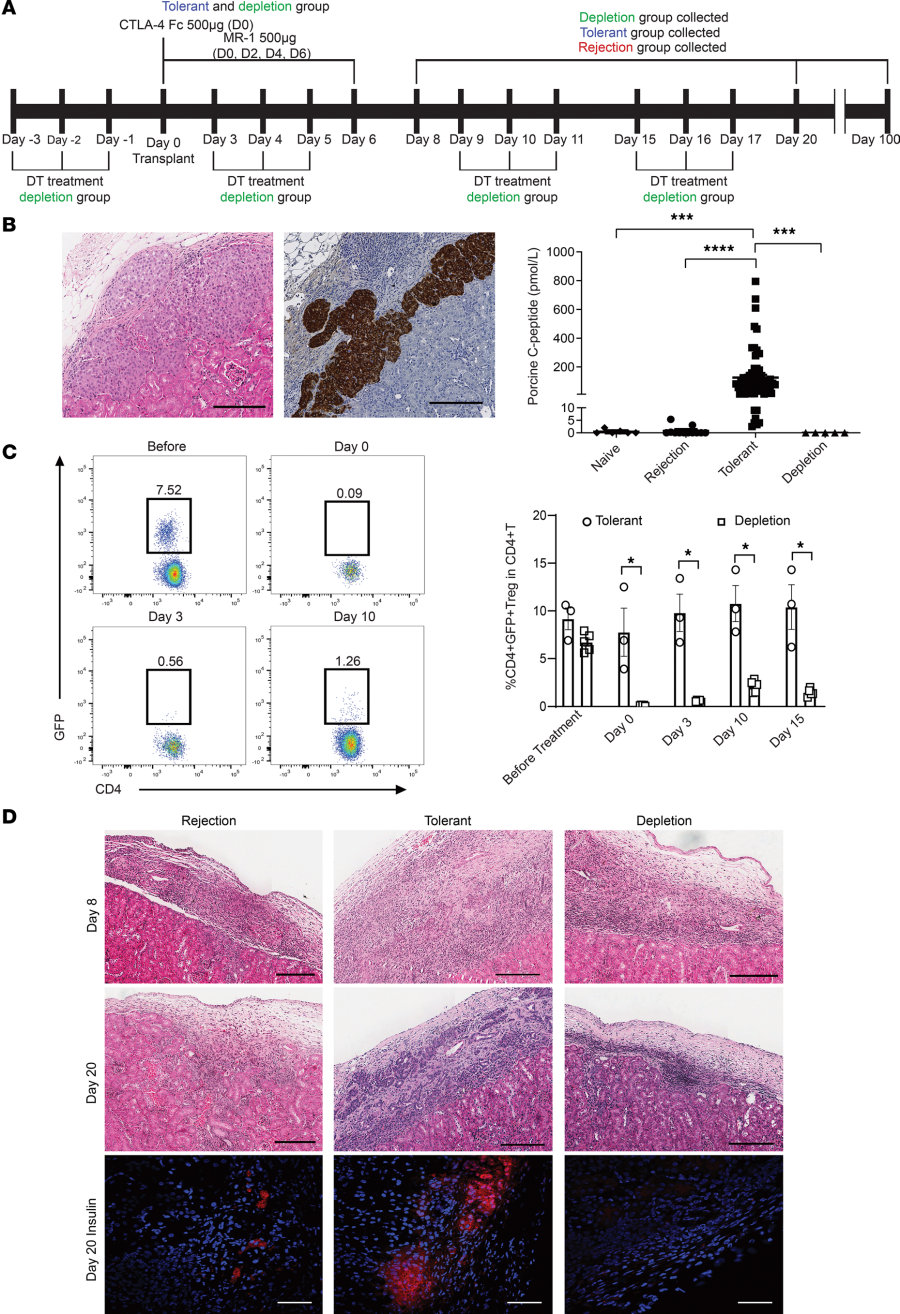
Depletion of Tregs in induction phase abolishes transplant tolerance induced by short-term treatment with CTLA4-Fc/MR1 in DEREG mouse recipients of porcine NICC grafts. (**A**) Schematic illustration of CTLA4-Fc/MR1 treatment (tolerant group) and Treg depletion (depletion group) timelines for DEREG recipients receiving porcine NICC transplants. DEREG recipients assigned to the rejection group did not receive any treatments. Samples were collected from all groups on days 8, 20, and ≥100 after transplantation. (**B**) Functional assessments of porcine NICC transplants in DEREG recipients day 100. Micrographs of H&E (left) and immunohistochemistry (IHC) insulin-positive staining (brown) (right) of porcine NICC grafts in the tolerant group are shown. Data for serum porcine C-peptide in tolerant group (*n* = 69), rejection group (*n* = 12), depletion group (*n* = 5), and control DEREG mice, which were neither transplanted nor treated with CTLA4-Fc/MR1 (naive group/mice) (*n* = 6). (**C**) Confirmation of Treg depletion in CTLA4-Fc/MR1–treated DEREG recipients with the DT injection regimen. Representative pseudocolor plots of CD4 versus GFP (gated on CD4^+^ T cells) showing CD4^+^GFP^+^ Tregs in peripheral blood mononuclear cells (PBMCs) from deletion group recipients before transplant and days 0, 3, and 10 after transplant. The proportion of CD4^+^GFP^+^ Tregs in CD4^+^ T cells of PBMCs in the depletion group (*n* = 5) and tolerant group (*n* = 3) before treatment and on day 0, day 3, day 10, and day 15. (**D**) Micrographs of H&E-stained porcine NICC grafts at day 8 and day 20 and immunofluorescence insulin-stained porcine NICC grafts day 20 in the rejection, tolerant, and depletion groups. The positive insulin staining was shown in red. H&E and IHC images were visualized using Aperio ImageScope (v.12.4.0.7018) software (Leica Biosystems). The immunofluorescence images were imaged on Olympus FV ≥1000 confocal microscope with FV10-ASW 4.2 software. Scale bar: 200 μm (**B** and H&E, **D**); 50 μm (immunofluorescence, **D**) Kruskal-Wallis test was used in **B**, and Mann-Whitney test was used in **C**. Data were from 9 independent experiments and shown as mean ± SEM. Label of statistical significance: *P < 0.05, ***P < 0.001, ****P < 0.0001.

To determine the role of Tregs in the induction of tolerance in this model, DEpletion of REGulatory T cells (DEREG) mouse recipients were transplanted with NICC under their renal capsules and received short-term treatment with CTLA4-Fc/MR1 to induce tolerance (Figure 1A). In mice treated with CTLA4-Fc/MR1 (tolerant group/mice), NICC grafts were prolonged beyond 100 days after transplantation with intact pig islets being surrounded by sparse immune cell infiltration (Figure 1B). Serum porcine C-peptide, indicating functional islets, was detected (125.6 ± 158.8 pmol/L, *n* = 69) in tolerant group mice at 100 days after transplantation while none was detected in the recipients without CTLA4-Fc/MR1 treatment (0.73 ± 1.64 pmol/L, *n* = 12) (rejection group mice) (*P* < 0.0001) or in control mice without transplantation and no treatment (naive group mice) (0.45 ± 0.73 pmol/L, *n* = 6) (*P* < 0.001) (Figure 1B).

Next, specific depletion of Tregs was performed in DEREG recipients at the time of CTLA4-Fc/MR1 treatment by administration of diphtheria toxin (DT) (depletion group) (Figure 1A). Treg (CD4^+^GFP^+^/Foxp3^+^) depletion was verified in the peripheral blood of DEREG recipients (Figure 1C). Histological examination of NICC grafts on day 8 showed immune cell infiltration under the kidney capsule in all groups (Figure 1D). By day 20, NICC grafts from recipients treated with CTLA4-Fc/MR1 had intact islets with positive insulin staining and were surrounded by a small cellular infiltrate (Figure 1D). In contrast, NICC graft sites in Treg-depleted recipients showed no intact islets and no insulin staining with immune cell infiltration, similar to that seen in rejected NICC grafts. Serum porcine C-peptide was not detected at day 100 in the depletion group, confirming NICC graft rejection (Figure 1B). Together, these results demonstrate that Tregs are essential for the induction of porcine NICC graft tolerance induced by CTLA4-Fc/MR1 treatment.

### Tregs expand systemically in the spleen and locally in the DLN of long-term tolerant recipients.

Having demonstrated previously the importance of Tregs in the maintenance of tolerance (17), we tracked T cells and Tregs in the spleen, axillary lymph node (ALN), and graft-draining lymph node (DLN) in DEREG recipients in rejection and tolerant groups at day 8 and day 100, as well as naive mice, using flow cytometry. No significant differences were found in the proportion of CD3^+^ T cells in these lymphoid organs between different groups at day 8 and day 100, in CD4^+^ and CD8^+^ T cells as a proportion of CD3^+^ T cells in each of the sites, or at different time points (Supplemental Figure 1, A–C; supplemental material available online with this article; https://doi.org/10.1172/jci.insight.169119DS1).

**Figure 2:**
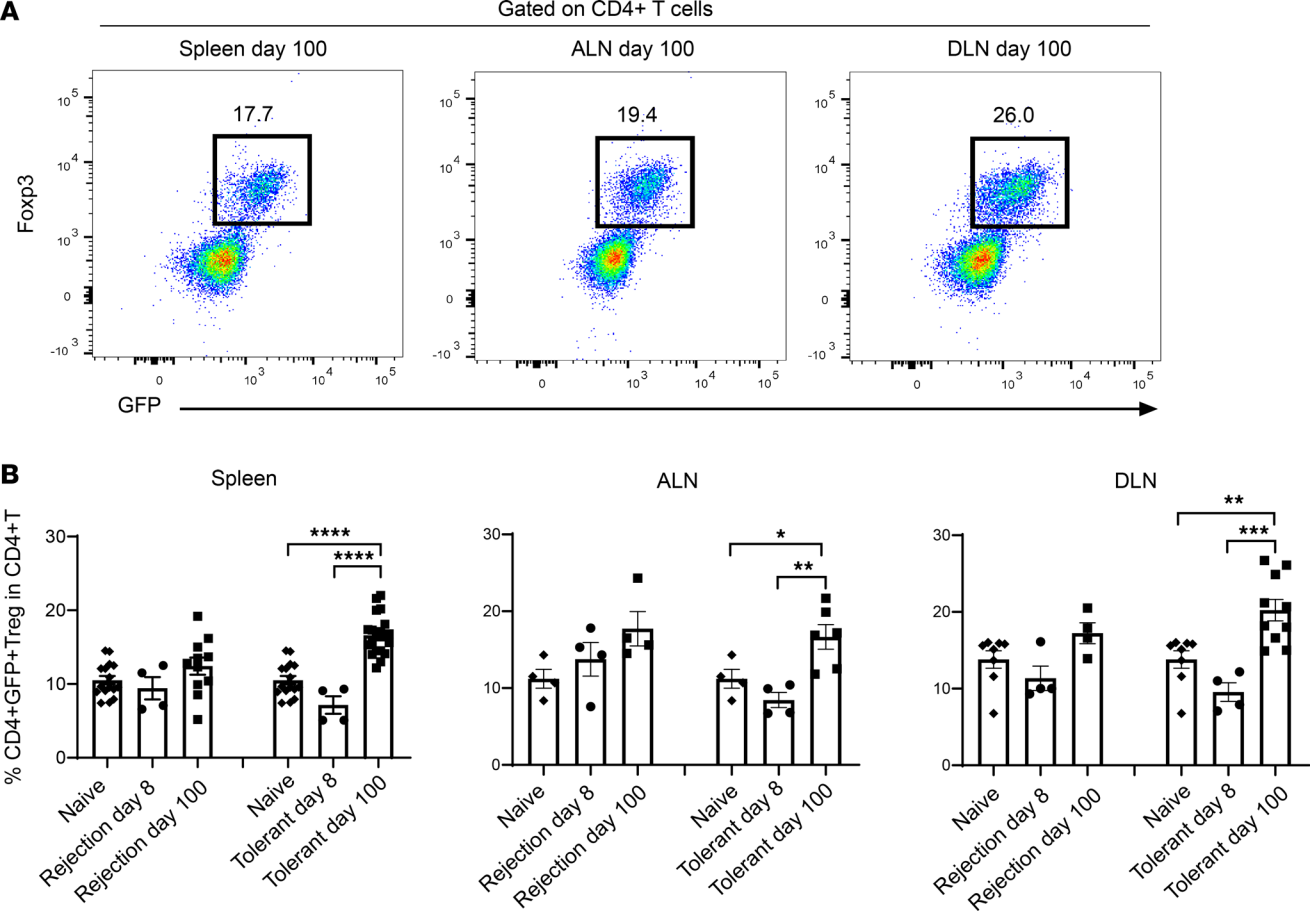
Tregs expand in spleen and lymph nodes of transplant-tolerant mice during transplantation. (**A**) Confirmation of surface GFP expression representing intracellular Foxp3 expression in DEREG recipients. Representative pseudocolor plots of GFP versus Foxp3 (gated on CD4^+^ T cells) revealed that CD4^+^GFP^+^ T cells were CD4^+^Foxp3^+^ Tregs in the spleen, axillary lymph node (ALN), and draining LN (DLN) in the tolerant group at day 100 after transplantation. (**B**) The proportions of CD4^+^GFP^+^Foxp3^+^ Tregs within CD4^+^ T cells of the spleen, ALN, and DLN in tolerant group (*n* = 4–20), rejection group (*n* = 4–11) on day 8 and day 100 after transplantation, and control naive group (*n* = 4–16) by flow cytometry analysis. The comparison within the group between different time points is shown. A 1-way ANOVA was used. Data were from 4 independent experiments and shown as mean ± SEM. *P < 0.05, **P < 0.01, ***P < 0.001, ****P < 0.0001.

Next, we examined the proportion of Tregs, based on the expression of GFP and Foxp3, in these lymphoid organs of day 100 DEREG recipients and verified CD4^+^GFP^+^ T cells were CD4^+^Foxp3^+^ Tregs (Figure 2A). Although no significant difference of Tregs in the lymphoid organs was found between rejection and tolerance at day 8 (Supplemental Figure 1D), within the tolerant group, there was a significant increase of Tregs in the spleen, ALN, and DLN between day 8 and day 100, indicating expansion of Tregs over time (Figure 2B). In contrast, there were no differences in Tregs in spleen, ALN, and DLN of mice in the rejection group over time (Figure 2B). Together, the data suggest that Tregs expand in the DLN and egress into the circulation, leading to increased numbers in the spleen and ALN during transplant tolerance.

### Expanded Tregs with enhanced CD127 expression in NICC grafts provide local suppression of activated CD4^+^ T cells.

**Figure 3:**
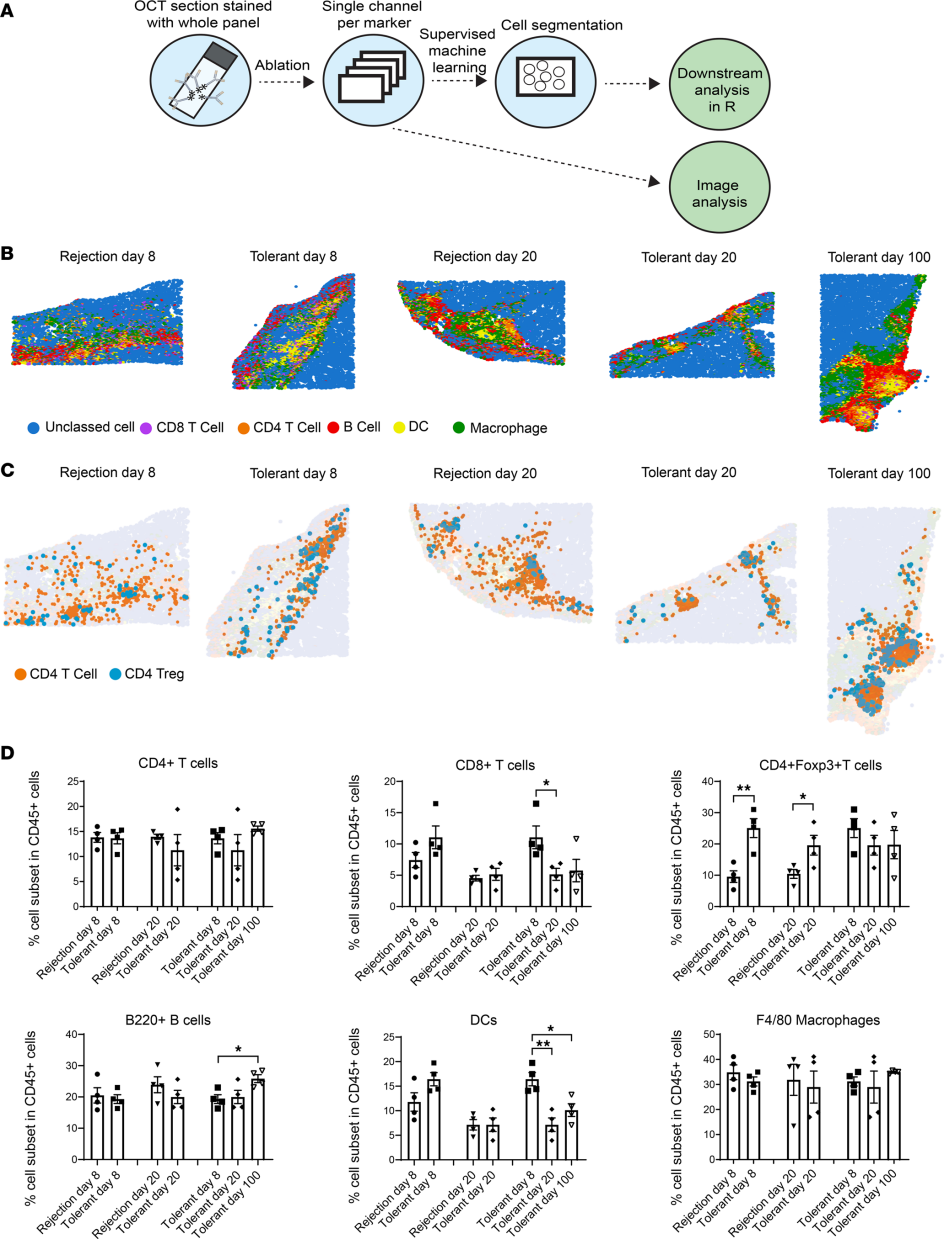
Immune cell classification in the graft site shows expanded Tregs within tolerant grafts. (**A**) A schematic image showing the imaging mass cytometry (IMC) staining and data analysis pipeline. (**B**) Presentation of CD8^+^ and CD4^+^ T cells, B cells, macrophages, and dendritic cells (DCs) in both rejection and tolerant grafts. Representative pseudo-images of tolerant (day 8, 20, 100) and rejection (day 8, 20) groups by IMC. Cell group classification included CD8^+^ T cells (purple: CD45^+^CD3^+^CD8^+^CD4^–^), CD4^+^ T cells (orange: CD45^+^CD3^+^CD4^+^CD8^–^Foxp3^–^), B cells (red: CD45^+^CD3^–^B220^+^), DCs (yellow: CD45^+^F4/80^–^IA/IE^+^CD11c^+^), macrophages (green: CD45^+^F4/80^+^), and unclassed cells (blue: CD45^–^). Pseudo-image plots represented tissue area of average 1.044 mm^2^ (SEM = 0.06 mm^2^). (**C**) IMC pseudo-images of the same representative graft sites as in **B** showing distribution of CD4^+^ T cells (orange) and CD4^+^ Tregs (blue: CD45^+^CD3^+^CD4^+^CD8^–^Foxp3^+^ cells). (**D**) Quantification of cell types between experimental groups/days based on classification in **B** and **C**, where numbers were presented as percentage of all CD45^+^ cells. An unpaired 2-tailed *t* test was used for comparing rejection group to tolerant group and spleens to grafts. A 1-way ANOVA was used for comparing day 8, day 20, and day 100 within tolerant group. Error bars indicate the mean ± SEM. Label of statistical significance: *P < 0.05, **P < 0.01.

As memory Tregs are found predominantly in tissue, we hypothesized that graft Tregs may be critical in suppressing the local immune response and inflammation within grafts of tolerant mice. Using imaging mass cytometry (IMC) (Figure 3A), we evaluated the differences between graft-specific infiltrating immune cell profiles in tolerant (day 8, 20, 100) and rejection groups (day 8, 20) at different time points. Pseudo-images using manual cell classification were generated using original single-cell *x* and *y* location to visualize immune cell distribution within the NICC graft sites (Figure 3, B and C). Overall, graft-infiltrating immune cells were identified in both rejecting and tolerant grafts with CD4^+^ and CD8^+^ T cells, B cells, DCs, macrophages, and Tregs all being present (Figure 3, B and C). The number of CD4^+^ and CD8^+^ T cells, B cells, macrophages, and DCs did not differ significantly between tolerant and rejection groups at day 8 and day 20 (Figure 3D). However, the number of Tregs was significantly higher in tolerant mice on day 8 and day 20 when compared with rejection mice, indicating increased numbers of Tregs within the graft at relatively early time points after CTLA4-Fc/MR1 treatment (Figure 3D). Moreover, Tregs were present in day 100 tolerant grafts. Apart from an increase in Tregs, CD8^+^ T cells were significantly decreased in tolerant mice at day 20 when compared with day 8, and DCs were decreased over time in tolerant mice (Figure 3D). Interestingly, an increase of B cells was observed in the NICC grafts of tolerant mice at day 100 when compared with day 8 grafts (Figure 3D). Further, the pseudo-images of a comparison between distribution of Tregs and CD4^+^Foxp3^–^ T cells visualized the relationship between the 2 subsets. While the Tregs and CD4^+^Foxp3^–^ T cells were scattered throughout the graft site in both tolerant and rejection groups at day 8 and day 20, by day 100 Tregs were surrounding and in direct contact with CD4^+^Foxp3^–^ T cells, forming a cell cluster within the tolerant graft (Figure 3C). These in vivo data are consistent with published in vitro data suggesting that Treg suppression involved cell-cell contact mechanisms within the graft (19) or may indicate the induction of a Foxp3^–^ regulatory population, such as type 1 regulatory T cells (20).

Within the grafts of tolerant mice, the percentages of CD25^+^CD4^+^Foxp3^–^ and CD127^+^CD4^+^Foxp3^–^ subsets decreased at day 20 and day 100 (both *P* < 0.05), when compared with day 8, suggesting less activation of CD4^+^Foxp3^–^ T cells (21) over time (Supplemental Figure 3A). Interestingly, the IA/IE^+^(MHC-II)CD4^+^ proportion within CD4^+^Foxp3^–^ T cells was higher within the grafts of tolerant mice, when compared with rejecting grafts at day 8 and day 20, and this proportion was maintained until day 100 (Supplemental Figure 2A). This suggests the ongoing presence of activated CD4^+^ T cells and is consistent with the persistence of xenoantigen-driven activation. However, there were no major differences within tolerant and rejecting grafts of the CD8^+^ T cell subpopulations (Supplemental Figure 2B). This is consistent with published data suggesting a limited role for CD8^+^ T cells in the cellular rejection of NICC grafts (22). There also were no differences for CD27^+^B220^+^B cells and IA/IE^+^F4/80^+^ macrophages between groups and time points (Supplemental Figure 2C).

**Figure 4:**
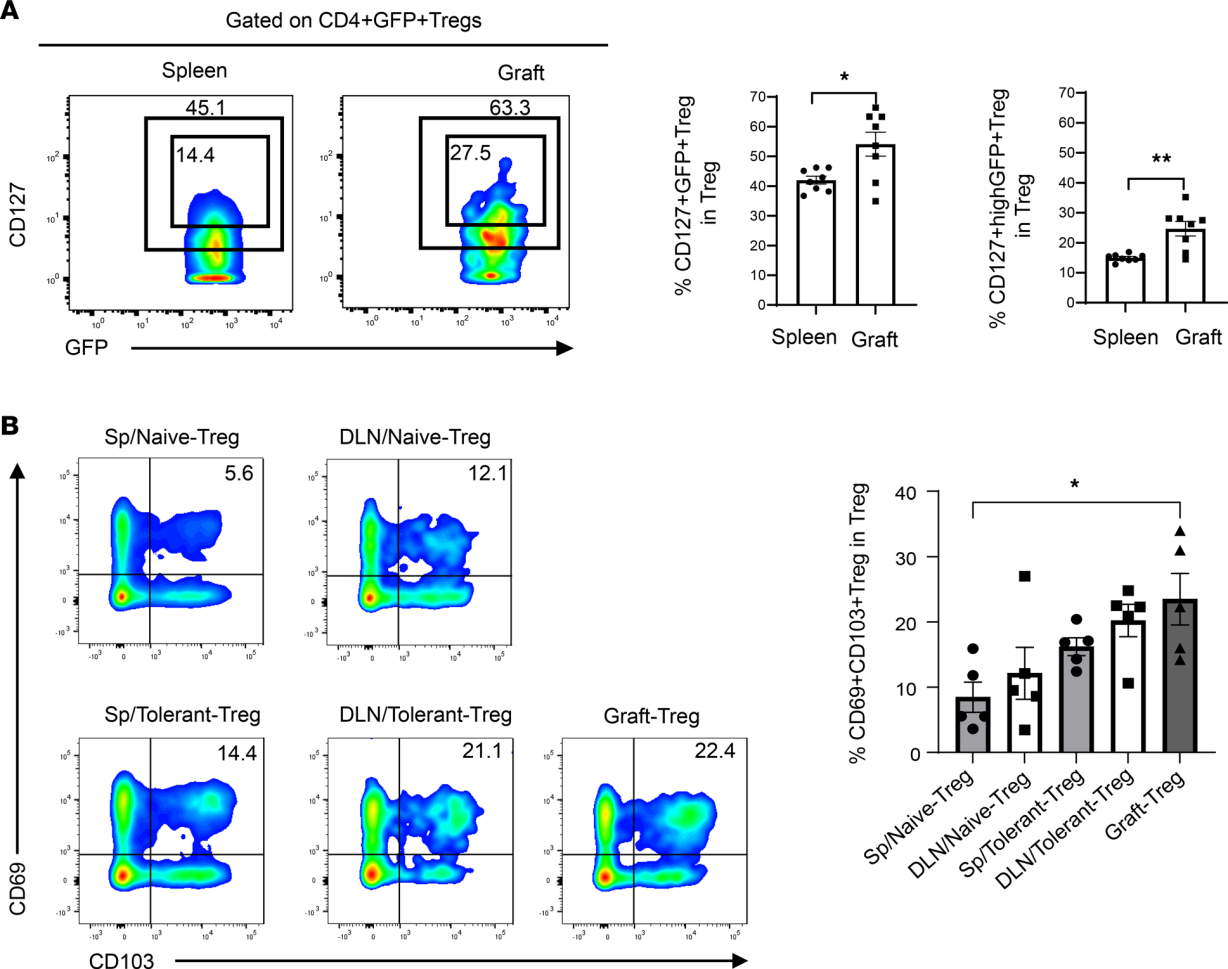
The features of Tregs within grafts in mouse recipients receiving CTLA4-Fc/MR1 treatment at 100 days after transplantation. (**A**) Proportions of CD127^+^ Tregs and CD127^hi^ Tregs within CD4^+^GFP^+^Foxp3^+^ Tregs of spleen (*n* = 8) and graft (*n* = 8) in tolerant group on day 100 after transplantation by flow cytometry analysis. (**B**) Proportion of CD69^+^CD103^+^ Tregs within CD4^+^GFP^+^Foxp3^+^ Tregs of the spleen, DLN, and graft in tolerant group (*n* = 5) and in naive group (*n* = 5) by flow cytometry analysis. An unpaired *t* test was used in **A**, and a 1-way ANOVA was used in **B**. Data were from 3 independent experiments and shown as mean ± SEM. *P < 0.05, **P < 0.01.

Skin Tregs have been shown to have increased expression of CD127, and CD127^hi^ has been proposed as a marker of murine Treg memory (10, 11). CD127 was assessed on Tregs within tolerant grafts at day 100. A high proportion of CD4^+^GFP^+^ Tregs within the tolerant grafts were CD127^hi^CD4^+^GFP^+^ Tregs (CD127^hi^ Tregs): 54.1% ± 4.0% (CD127^+^) and 24.6% ± 32.4% (CD127^hi^) of graft Tregs versus 41.9% ± 1.3% (CD127^+^) and 14.9% ± 0.4% (CD127^hi^) of splenic Tregs (Figure 4A). These data identify such memory-like CD127^hi^ Tregs residing in the tolerant graft of mice recipients. This was supported by the finding that, when compared with naive Tregs, the proportion of Tregs coexpressing CD69 and CD103 was significantly elevated in the grafts of tolerant mice at least 100 days after transplant (Figure 4B). The coexpression of CD69 and CD103 is a marker of tissue-resident Tregs and is thought to prevent egress from resident tissues (23, 24).

Taken together, these data support the hypothesis that there was ongoing CD4^+^ T cell activation within NICC grafts, which was under a state of continuous suppression by antigen-experienced and activated effector/memory-like Tregs that resided within the graft. This suggests that CD127^hi^ tissue Tregs are important for the ongoing acceptance of the graft in transplant tolerance.

### Memory-like CD127^hi^ Tregs exist in the spleens of tolerant mouse recipients.

**Figure 5:**
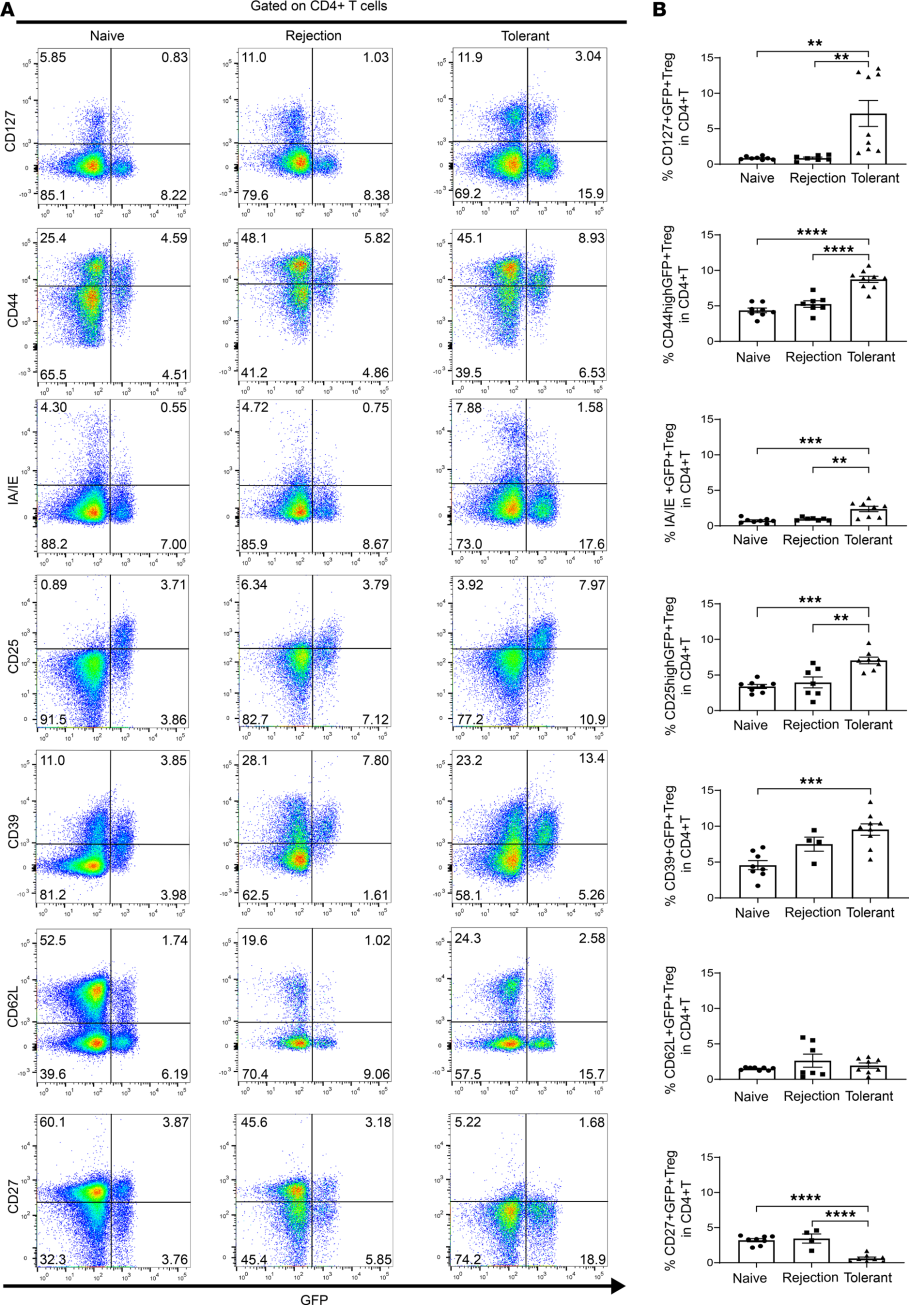
Expanded splenic Tregs show memory-like Treg phenotype in tolerant mouse recipients. (**A**) Representative pseudocolor plots of GFP versus various immune markers: CD127, CD44, IA/IE, CD25, CD39, CD62L, or CD27 (gated on CD4^+^ T cells) in spleens of tolerant group, rejection group at day 100 after transplantation, and control naive group by flow cytometry analysis. (**B**) Proportions of CD127^+^GFP^+^, CD44^hi^GFP^+^, IA/IE^+^GFP^+^, CD25^hi^GFP^+^, CD39^+^GFP^+^, CD62L^+^GFP^+^, and CD27^+^GFP^+^ Tregs in total CD4^+^ T cells in spleens of rejection group (*n* = 4–7) and tolerant group (*n* = 8–9) day 100 after transplantation and naive group (*n* = 8). A 1-way ANOVA was used. Data were from 3 independent experiments and shown as mean ± SEM. **P < 0.01, ***P < 0.001, ****P < 0.0001.

In the context of transplant tolerance, naive Tregs are exposed to donor antigen in secondary lymphoid organs where they are activated, proliferate, and differentiate into effector Tregs that are antigen specific with potent suppressive function that protects the graft from rejection (25, 26). Currently it is not clear whether these effector Tregs transition to memory Tregs in secondary lymphoid organs or within the graft (4, 11). Therefore, in-depth profiling of immune expression panels to determine existence and phenotype of memory Tregs was undertaken. CD4^+^GFP^+^/Foxp3^+^ Tregs were assessed by flow cytometry panels for their expression of CD44, CD127, CD62L, MHC-II, CD27, CD25, and CD39, which are markers of memory Tregs as described by others (Supplemental Figure 3A). There was a significant increase of CD127^+^GFP^+^, CD44^hi^GFP^+^, IA/IE^+^GFP^+^, CD25^hi^GFP^+^, and CD39^+^GFP^+^ Treg subpopulations in the spleen of tolerant mice, when compared with naive and/or rejection group animals (Figure 5). Meanwhile there was no significant increase in CD62L^+^GFP^+^ Tregs (Figure 5) but a significant increase of CD62L^–^GFP^+^ Tregs in the spleen of tolerant mice when compared with both naive and rejection groups (Supplemental Figure 4A). Further analysis within the CD4^+^GFP^+^ Treg population verified that this was predominantly due to an increase of CD127^hi^GFP^+^ Tregs and MHC-II^+^GFP^+^ Tregs whereas the other Treg subsets (CD25^hi^GFP^+^, CD44^hi^GFP^+^, CD39^+^GFP^+^) were similar across all groups (Supplemental Figure 4B). The presence of a high proportion of CD127^hi^ Tregs in the spleen of tolerant mice suggests that memory-like Tregs may migrate among the graft, secondary lymphoid organs, and circulation in the context of transplant tolerance.

### Treg heterogeneity is based on activation status and anatomical location.

**Figure 6:**
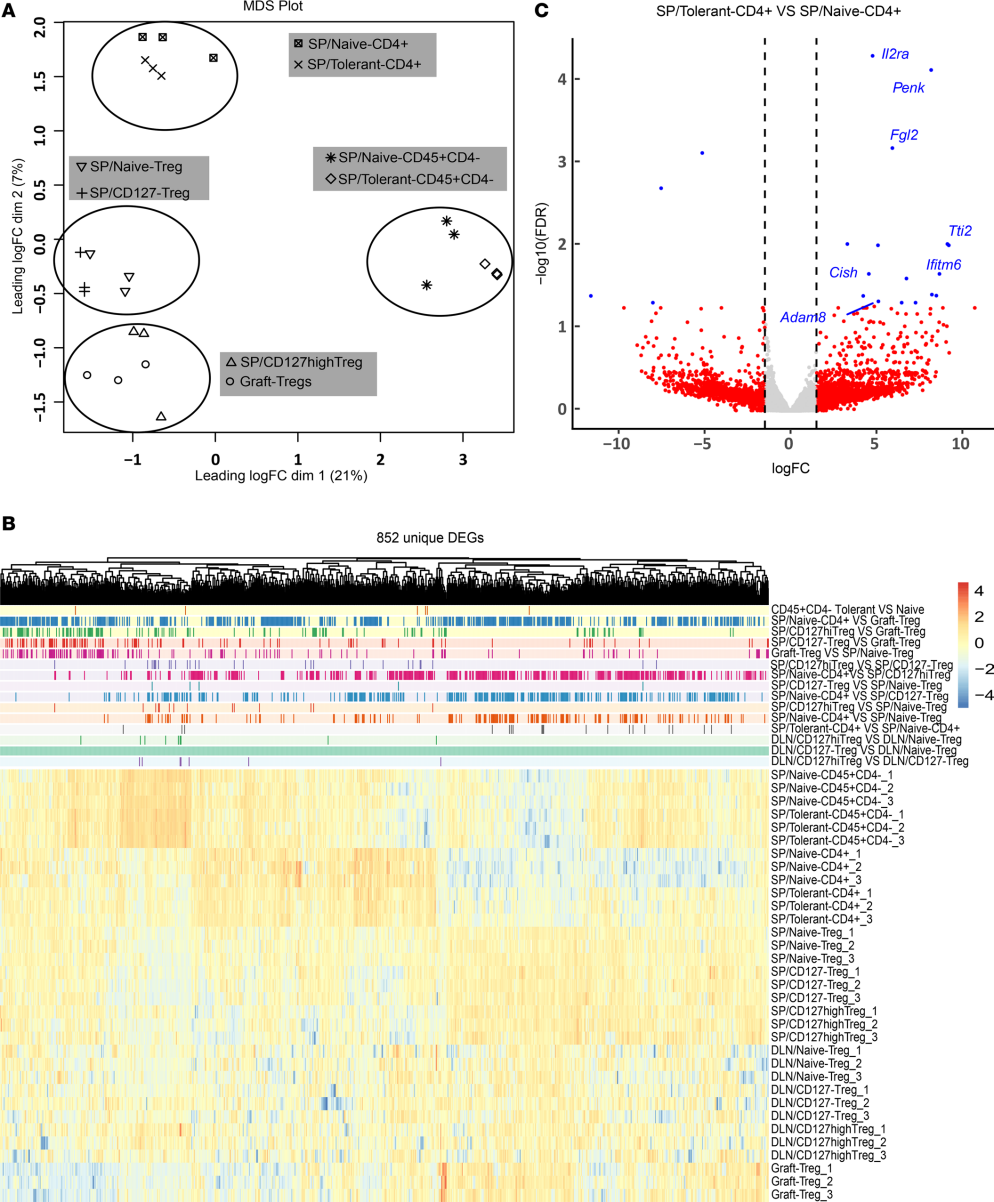
Distinct transcriptional profiles of Foxp3^–^ CD4^+^ T cell and Treg subsets between transplant-tolerant mice and naive mice. (**A**) Multidimensional scaling analysis (MDS) of bulk RNA-Seq showed the segregation of Tregs and Foxp3^–^ immune cells. Treg subsets comprised splenic CD127^+/hi^CD4^+^GFP^+^ Treg (SP/CD127^hi^ Treg) and CD127^–/lo^CD4^+^GFP^+^ Treg subsets (SP/CD127^–^ Treg) and graft-infiltrating CD4^+^GFP^+^ Tregs (graft Tregs) from tolerant group day 100 after transplantation, as well as CD4^+^GFP^+^ Tregs from spleen of naive mice (SP/Naive Tregs). The Foxp3^–^ subsets included CD4^+^GFP^–^ T cells and CD45^+^CD4^–^ immune cells from spleens of naive mice (SP/Naive CD4^+^ and SP/Naive CD45^+^CD4^–^) and spleens of tolerant mice (SP/Tolerant CD4^+^ and SP/Tolerant CD45^+^CD4^–^). (**B**) Heatmap of the 852 unique DEGs (FDR < 0.05) among the 1,740 DEGs derived from 15 paired cross-comparisons. These cross-comparisons were listed in Supplemental Table 1. (**C**) The volcano plot showed DEGs in SP/Tolerant CD4^+^ subset compared with SP/Naive CD4^+^ subset. Vertical dashed lines on the volcano plot indicated a fold-change of ± 1.5, and DEGs with FDR < 0.05 were indicated in blue dots. Bulk RNA-Seq sample size for each cell subset was 3 samples (a pool of 3–4 mice/sample) in which DEREG recipients of tolerant group (*n* = 10, male mice) were from 2 independent transplant experiments, and DEREG naive mice (*n* = 6, 3 male mice for spleen samples, and 3 female mice for LN samples) were used.

To investigate Treg heterogeneity in this transplant tolerance model and impacts of CTLA4-Fc/MR1 treatment on immune cells, we investigated the transcriptomes of 7 Treg subsets and 4 Foxp3^–^ subsets, using bulk RNA-Seq. Treg subsets included Tregs (CD4^+^GFP^+^/Foxp3^+^) from infiltrating cells of tolerant grafts (graft Tregs) and the spleen (SP/naive Tregs) and DLN (DLN/naive Tregs) of naive mice and CD127^hi^ Tregs and CD127^–/lo^ Tregs from the spleen and DLN of tolerant mice (Supplemental Figure 5); and Foxp3^–^ subsets included CD4^+^GFP^–^ cells and CD45^+^CD4^–^ cells from spleens of tolerant and naive mice. The multidimensional scaling (MDS) analysis demonstrated that all Treg subsets, CD4^+^Foxp3^–^ T subsets, and CD45^+^CD4^–^ cell subsets clearly separated (Figure 6A); the plot of Foxp3 versus GFP gene expression verified a regulatory phenotype for all Treg subsets (Supplemental Figure 6). Next, using a false discovery rate (FDR) less than 0.05, 15 pairwise comparisons (described in Supplemental Table 1) identified 852 unique differentially expressed genes (DEGs) that clearly distinguished CD45^+^CD4^–^ T cells, Foxp3^–^CD4^+^ T cells, and Treg subsets, with no striking differences found for CD45^+^CD4^–^ cells isolated from naive and tolerant animals (Figure 6B). There were minor differences in splenic Foxp3^–^CD4^+^ T cells between naive and tolerant groups, and the most notable differences were shown across the Treg subsets (Figure 6B). The upregulated DEGs of *Il12ra* (27) and *Penk* (28) on CD4^+^Foxp3^–^ T cells of the tolerant group suggested that CTLA4-Fc/MR1 treatment did not fully inhibit activation of conventional CD4^+^ T cells (Figure 6C). Interestingly, fibrinogen-like protein 2 (*Fgl2*), which is reported to be preferentially expressed on memory T cells with the presence of IFN-γ (29), was upregulated on CD4^+^Foxp3^–^ T cells of the tolerant group (Figure 6C). FGL2 is reported to have a positive correlation with T cell immunoglobulin mucin receptor 3 (TIM3) and CTLA4 (30) and is an effector molecule that promotes Treg activity (29, 31).

**Figure 7:**
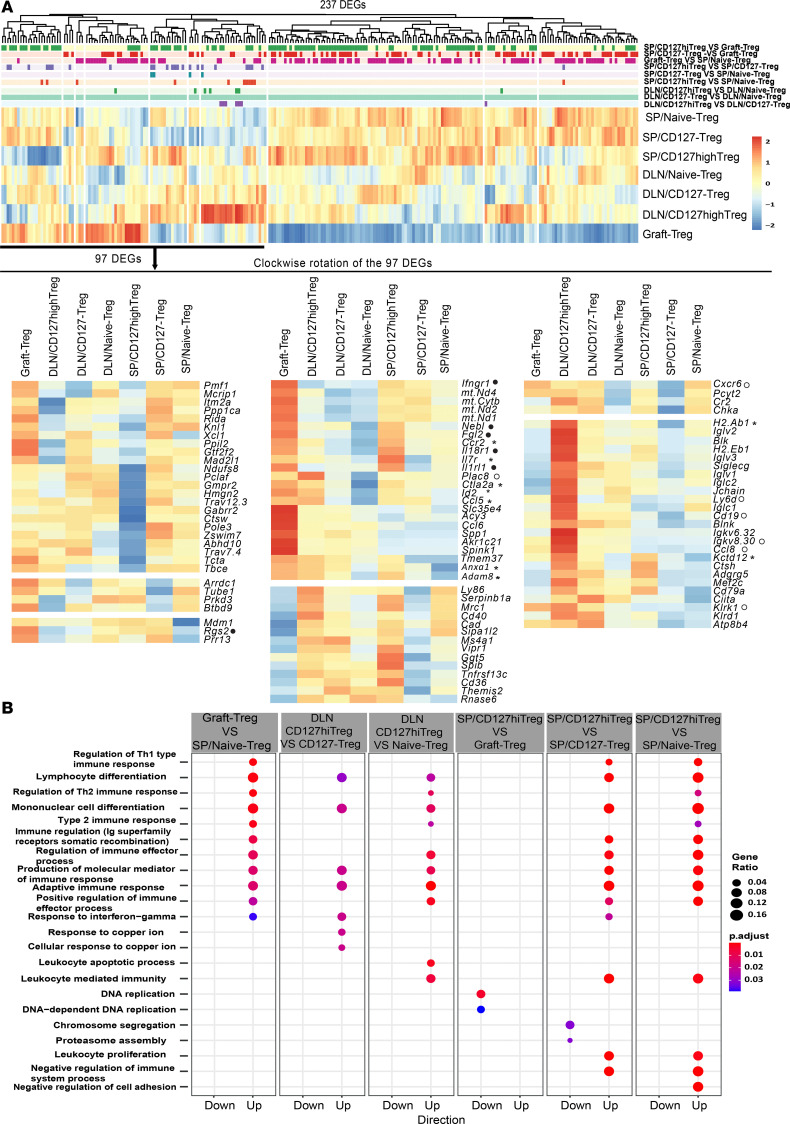
CD127^hi^ Tregs and graft Tregs in transplant-tolerant recipients share transcriptional trajectory with tissue Tregs. (**A**) Heatmap of the 237 unique DEGs (FDR < 0.05) derived from 9 paired cross-comparisons within 7 Treg subsets (Supplemental Table 1 with a symbol, ^§^). Treg subsets were the same as shown in Figure 6. Compared with either naive Treg or CD127^–^ Treg subsets, the shared upregulated DEGs or tendency of enhanced gene expressions (DEGs with FDR < 0.05 for at least 1 paired comparison) across SP/CD127^hi^ Treg, DLN/CD127^hi^ Treg and graft Treg subsets were marked with the star, among DLN/CD127^hi^ Treg and graft Treg subsets were indicated with the filled dot, and among SP/CD127^hi^ Treg and graft Treg subsets were marked with the unfilled dot. (**B**) GO Biological Process pathway analysis based on DEGs (absolute log FC > 1.5 and *P* < 0.01) in the 6 paired cross-comparisons listed in Supplemental Table 3.

Next, we focused on DEGs between different Treg subsets and identified 427 DEGs (FDR < 0.05) (Supplemental Table 1), including 158 overlapping DEGs and 269 DEGs that were unique to specific Treg subsets. After we removed 32 genes that were uninformative to the process, the heatmap of the 237 DEGs showed large differences in gene expression between graft Tregs and Treg subsets of the spleen or DLN (Figure 7A). There were moderate differences between splenic Treg and DLN Treg subsets and minor differences within the 3 Treg subsets of the spleen and DLN (Figure 7A). These data verified a Treg heterogeneity among Treg populations within transplant-tolerant mice and differed from that seen in naive mice. When looked at in the context of the MDS data (Figure 6A), these gene profiles showed that the Treg populations from transplant-tolerant mice shared many signaling pathways in common but also had different transcriptional profiles based on their anatomical location and activation status.

### Lymphoid memory-like CD127^hi^ Tregs and graft Tregs of tolerant mice show a shared transcriptional trajectory with tissue Tregs.

Next, we explored possible precursor relationships between secondary lymphoid organ Tregs and nonlymphoid tissue Tregs. The MDS showed naive Treg and CD127^–/lo^ Treg subsets tended to subcluster together; meanwhile CD127^hi^ Treg and graft Treg subsets tended to group together, indicating a similarity between lymphoid CD127^hi^ Tregs and graft Tregs in transplant tolerance (Figure 6A). This also supports the hypothesis that the graft Treg subset was the result of further differentiation of lymphoid Treg populations. We next interrogated the DEGs in splenic CD127^hi^ Treg, DLN CD127^hi^ Treg, and graft Treg subsets compared with either naive Treg or CD127^–/lo^ Treg subsets. A summary of selected upregulated DEGs and their identified functions or associations is outlined in Supplemental Table 2. The genes expressed by the subsets included shared upregulated DEGs or those with a tendency for enhanced gene expression (DEGs with FDR < 0.05 for at least 1 paired comparison) across the 3 Treg subsets, including *Il7r* (*CD127*), *Kctd12*, *H2*.*Ab1*, *Ctla2a*, *Anxa1*, *Adam8*, *Ccr2*, *Id2*, and *Ccl5* (marked *); the DEGs among DLN CD127^hi^ Tregs and graft Tregs included *Klrk1*, *Ccl8*, *Cxcr6*, *Ly6d*, *Plac8*, *CD19*, and *Igkv8*.*30* (unfilled dot); and DEGs among splenic CD127^hi^ Tregs and graft Tregs included *Nebl*, *Fgl2*, *Rgs2*, *Il1rl*, *Il18r1*, and *Ifngr1* (filled dot) (Figure 7A and Supplemental Figure 7). The enhanced *Il7r* expression on graft Tregs correlated well with the increased proportion of CD127^hi^ Tregs found in the grafts (Figure 4A). The majority of these DEGs have been identified on activated/effector or memory Tregs, or tissue Tregs found in various tissues or tumors, and demonstrated high suppressive function or tissue repair in numerous animal models and clinical studies (Supplemental Table 2). For instance, splenic CD127^hi^ and graft Tregs expressed *Il1rl1*, which encodes ST2/IL33 receptor. ST2^+^ tissue Tregs are highly suppressive, are associated with homeostasis and tissue repair function, and are found in a broad range of tissues, including skin, muscle (32), colon/intestine (33, 34), lung (33), brain (35), visceral adipose tissue (6, 33, 36), and kidney (37). The transcriptional regulator *Id2* has been shown to be essential for tissue-resident Tregsʼ differentiation, survival, and function (38).

To determine the biological processes specific to lymphoid CD127^hi^ Treg and graft Treg subsets, we tested DEGs on 6 paired cross-comparisons for enrichment of Gene Ontology (GO) terms (Biological Process, Benjamini-Hochberg–corrected *P* < 0.05; based on hypergeometric distribution against all observed genes) (Supplemental Table 3). This analysis showed a preponderance of pathways associated with immune regulation and revealed enrichment of upregulated DEGs in the IFN-γ pathway of graft Tregs and CD127^hi^ Tregs of the spleen and DLN (Figure 7B).

To verify the findings of DEGs, we further assessed by real-time reverse transcriptase polymerase chain reaction (RT-PCR) the cytokine expression of IL-2, IL-7, and IL-33 and TIM3, a coinhibitory receptor expressed on IFN-γ–producing T cells and Tregs (39). A significantly increased expression of *Il2* and *Il33* in day 8 grafts and *Il7* in day 100 grafts was observed in tolerant mice, but not rejecting mice, when compared with naive mice. Meanwhile there was a significant increase of *Havcr2* (*Tim3*) in both day 8 and day 100 grafts of tolerant mice, indicating effector T cell exhaustion (40) and immune regulation of Tregs (39) at early and late time points after transplantation (Supplemental Figure 8A). Expression of *Il7*, *Il33*, and *Havcr2* (*Tim3*) was enhanced significantly in the spleen of tolerant mice at day 100 after transplantation (Supplemental Figure 8B). This verifies the RNA-Seq data for *Il7r*, *Il1rl*, *Fgl2*, and *Il12ra* within tolerant mice.

Together, these data support the hypothesis that antigen-experienced Tregs are primed in the DLN to be effector and memory-like CD127^hi^ Tregs and migrate back to the graft, where a proportion take on the function and phenotype of memory-like tissue-specific Tregs.

### IL-7 leads to strong phosphorylation of STAT5 in CD127^hi^ Tregs.

**Figure 8:**
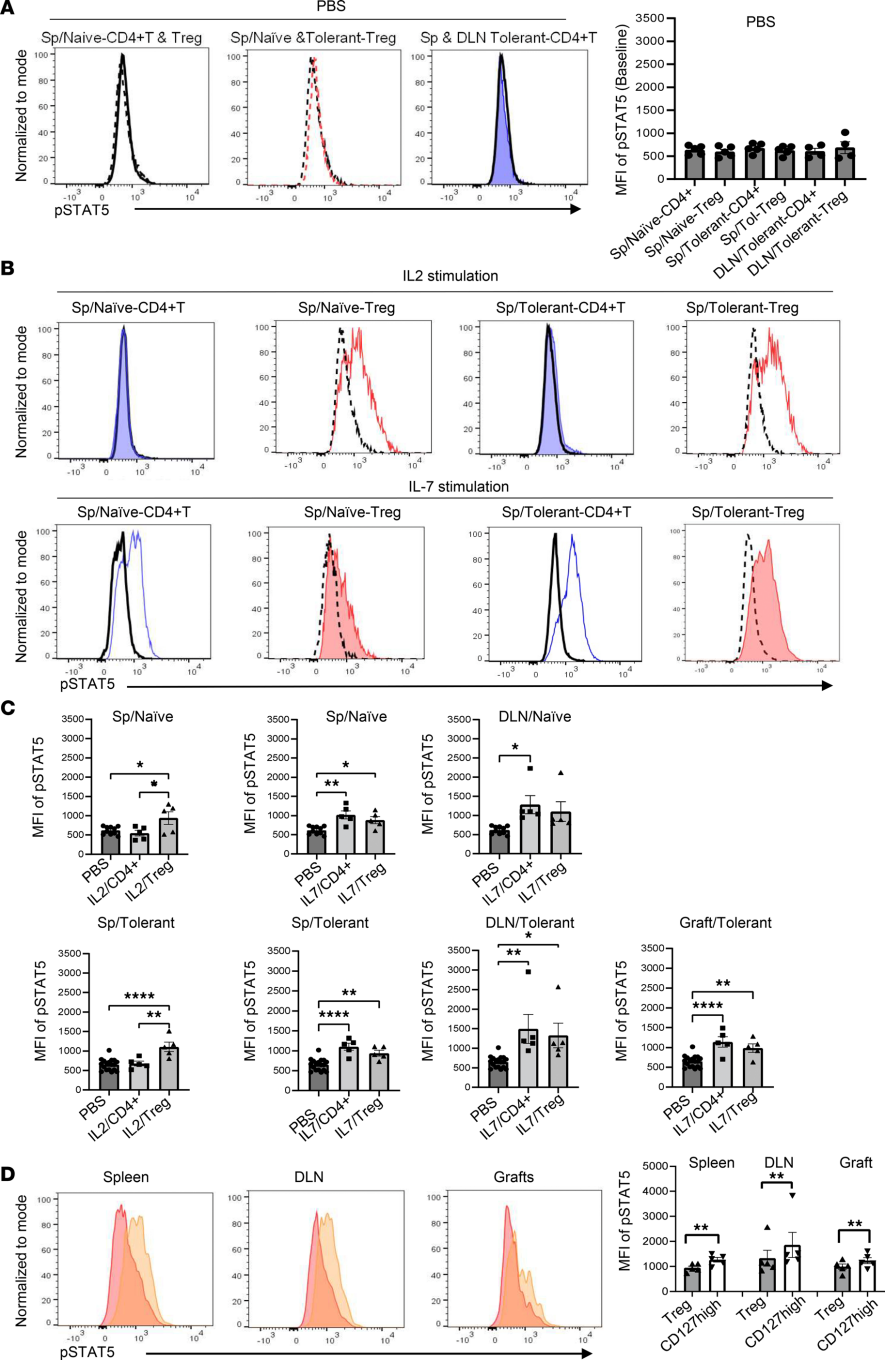
IL-7 stimulation induces enhanced phosphorylation of STAT5 in CD127^hi^ Tregs. (**A**) Baseline mean fluorescence intensity (MFI) of STAT5 phosphorylation (p-STAT5) on Tregs (gated on CD4^+^GFP^+^) and Foxp3^–^ CD4^+^ (gated on CD4^+^GFP^–^ cells) of the spleen and/or DLN from mouse recipients receiving CTLA4-Fc/MR1 treatment at day 100 (*n* = 5) and naive mice (*n* = 5) under PBS is shown. (**B**) Representative histograms of pSTAT5 expression on splenic CD4^+^GFP^–^ cells (blue shade — naive group, blue line — tolerant group) and CD4^+^GFP^+^ Tregs (red line — naive group, red shade — tolerant group), induced by IL-2 or IL-7 stimulation, and PBS (black line). (**C**) MFI of p-STAT5 expression on Tregs (*n* = 5) and CD4^+^GFP^–^ cells (*n* = 5) from the spleen, DLN, and/or graft of tolerant mice (*n* = 5) and naive mice (*n* = 5) induced by IL-2 or IL-7 stimulation compared with the baseline under PBS (*n* = 10, including Tregs and CD4^+^GFP^–^ cells in naive mice; and *n* = 18, including Treg and CD4^+^GFP^–^ cells in tolerant mice). (**D**) IL-7–induced p-STAT5 on Tregs (red) and CD127^hi^ Tregs (yellow) of spleens, DLNs, and grafts in tolerant mice is shown. A 1-way ANOVA was used in **A** and **C**, and the paired 2-tailed *t* test was used in **D**. Data were from 5 independent experiments and shown as mean ± SEM. *P < 0.05, **P < 0.01, ****P < 0.0001.

IL-2 is essential for naive Treg thymic development and is the main cytokine for homeostasis of peripheral naive/resting Tregs. IL-2 signals are propagated, in part, via activation of STAT5 (a positive regulator of IFN-γ production) (41), which functions as a key regulator of CD4^+^ T cell gene programming (42) and plays a critical role in Treg differentiation and function. IL-7 contributes to host defense by regulating the development and homeostasis of immune cells, including T cells (43). To understand the role of IL-2 and IL-7 on Tregs, using intracellular phosphor-protein staining with a multicolor flow cytometry panel, phosphorylation of STAT5 after IL-2 and IL-7 stimulation was assessed on Tregs (CD4^+^GFP^+^) and CD4^+^Foxp3^–^ T cells in recipient mice treated with CTLA4-Fc/MR1 at 100 days after transplantation. The baseline mean fluorescence intensity (MFI) of STAT5 phosphorylation was not different between cell types (Treg or CD4^+^GFP^–^ cell) in the 2 groups (transplant or naive mice), regardless of origin from the spleen or DLN (Figure 8A). As expected, a low dose of IL-2 (concentration 320 ng/mL) induced a vigorous phosphorylation of STAT5 in splenic CD4^+^GFP^+^ Tregs of naive mice but not CD4^+^GFP^–^ T cells (Figure 8, B and C). This IL-2–induced STAT5 phosphorylation was also observed on CD4^+^GFP^+^ Tregs in tolerant mice at 100 days. Interestingly, although splenic CD4^+^Foxp3^–^ T cells of tolerant mice showed increased expression of *Il2ra*, there was no increase in IL-2–induced STAT5 phosphorylation on splenic CD4^+^Foxp3^–^ T cells of tolerant mice (Figure 8, B and C). As expected, low-dose IL-7 (concentration 5 ng/mL) significantly induced STAT5 phosphorylation on CD4^+^GFP^–^ T cells in both naive mice and transplant recipient mice. Importantly, a similar level of STAT5 phosphorylation was induced in CD4^+^GFP^+^ Tregs by IL-7 stimulation in both naive mice and tolerant mice. When IL-7–induced STAT5 phosphorylation was evaluated on CD127^hi^ Tregs, a significant increase in STAT5 phosphorylation was seen in CD4^+^GFP^+^CD127^hi^ Tregs from the spleen, DLN, and graft of tolerant mice (Figure 8D). These data provide an explanation for the observed increase in Tregs within day 8 grafts, where there was high *Il2* expression, and the increased number of CD127^hi^ Tregs in grafts and spleens of tolerant mice on day 100, where there was high *Il7* expression (Figure 4A, Figure 5, and Supplemental Figure 8).

### Memory-like CD127^hi^ Tregs prevent xenograft rejection.

**Figure 9:**
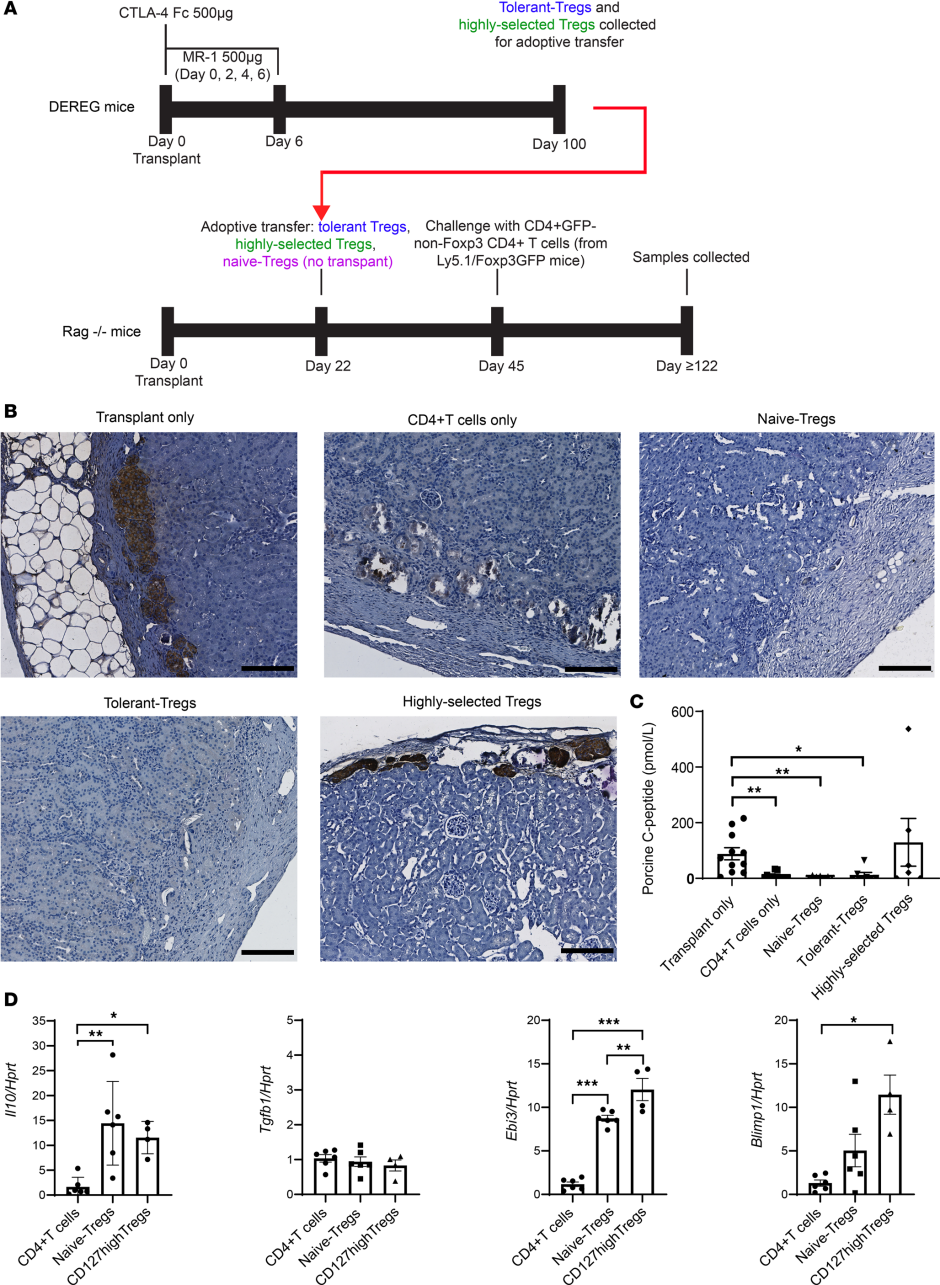
The suppressive function of memory-like CD127^hi^ Tregs is potent in vivo. (**A**) Schematic illustration of procedures and timelines for assessing suppressive function of Tregs in Rag^–/–^ mice receiving porcine NICC transplantation. DEREG mice were given CTLA4-Fc/MR1 described in Figure 1A. On ≥100 days after transplant, the CD4^+^GFP^+^ Tregs (tolerant Tregs) and CD127^hi^ Tregs from tolerant mice, as well as control CD4^+^GFP^+^ Tregs from naive mice (naive Tregs), were sorted and collected. Rag^–/–^ mice that received porcine NICC grafts were adoptively transferred with the tolerant Tregs, CD127^hi^ Tregs, or naive Tregs at day 22 after transplant. The 3 Treg adoptive transfer Rag^–/–^ mice groups and a Rag^–/–^ group that did not receive Tregs (CD4^+^ T cells only) were challenged with Foxp3^–^ CD4^+^GFP^–^ T cells from Ly5.1Foxp3^GFP^ mice on day 45 after transplant at 1:3 ratio (Treg/CD4^+^ T cell). The samples of these 4 groups and Rag^–/–^ mice with grafts and without transferring/challenging cells (transplant only) ≥120 days after transplant were collected. (**B**) Representative insulin-stained IHC images of porcine NICC grafts (brown as positive insulin staining) in Rag^–/–^ mice: 5 experimental groups. Scale bar: 150 μm. (**C**) Serum porcine C-peptide measurement for Rag^–/–^ mice experimental groups, including transplant only (*n* = 11), CD4^+^ T cells only (*n* = 12), naive Tregs (*n* = 7), tolerant Tregs (*n* = 7), and CD127^hi^ Tregs (*n* = 6). (**D**) Real-time RT-PCR performing measurement of *Il-10*, *Tgf-β*, *Ebi3*, and *Blimp1* on sorted splenic CD127^hi^CD4^+^GFP^+^Foxp3^+^ Tregs from tolerant mice day 100 after transplantation (CD127^hi^ Tregs) (*n* = 4), sorted splenic CD4^+^GFP^–^ T cells (CD4^+^ T cells) (*n* = 6), and CD4^+^GFP^+^Foxp3^+^ Tregs from naive mice (naive Tregs) (*n* = 6). Kruskal-Wallis test was used for the comparisons of serum porcine-C-peptide in **C**. A 1-way ANOVA followed by Tukey’s multiple comparison was used in **D**. Data were collected from 5 independent experiments and shown as mean ± SEM. *P < 0.05, **P < 0.01, ***P < 0.001.

To verify CD127^hi^ Treg suppressive ability, we sorted CD127^hi^ Tregs (CD127^hi^ CD44^+^CD62L^–^CD4^+^GFP^+^ Tregs) and tolerant Tregs (CD4^+^GFP^+^ Tregs) from the spleens of tolerant mice at ≥100 days after transplantation and adoptively transferred them into immune-deficient Rag^–/–^ recipients of NICC grafts (Supplemental Figure 9A). Sorted CD4^+^GFP^+^ Tregs from DEREG naive mice (naive Tregs) and CD4^+^GFP^–^ T cells (Foxp3^–^ CD4^+^ T) from Ly5.1Foxp3^GFP^ (CD45.1) naive mice were used as controls. First, the number of CD4^+^ T cells (Foxp3^–^) required for rejection of NICC grafts was determined after reconstitution of Rag^–/–^ recipients (Supplemental Table 4 and Supplemental Figure 9B). As described in Figure 9A, Tregs (CD45.2) were transferred into Rag^–/–^ recipients of NICC grafts 22 days after transplantation, and these mice were challenged with CD4^+^GFP^–^ T cells (CD45.1) at the ratio of 1:3 (Treg/CD4^+^ T) at day 45 and assessed for graft viability at day 122 or later. Insulin-positive islets were seen in recipient mice that received the memory-like CD127^hi^ Tregs and the insulin-positive control transplant–only group, not in those recipients that received unselected Tregs from tolerant mice or unselected naive Tregs or the Foxp3^–^ CD4^+^ T cell–only groups (Figure 9B). Islet function was further verified where the level of serum porcine C-peptide was not different between memory-like CD127^hi^ Tregs and the control transplant–only groups (Figure 9C). In contrast, no serum porcine C-peptide was detected in mice that received unselected Tregs from tolerant mice or naive mice (Figure 9C). The presence of both the memory-like CD127^hi^ Tregs (CD45.2) and the Foxp3^–^ CD4^+^ T cells (CD45.1) in the peripheral blood of transplanted Rag^–/–^ mice was verified 72 days after adoptive transfer of the Tregs (Supplemental Figure 9C). Here we demonstrated that memory-like CD127^hi^ Tregs prevented the NICC grafts from rejection in vivo and were more effective than naive or tolerant Tregs at preventing rejection.

### IFN-γ and regulatory cytokines, particularly IL-35 and IL-10 from CD127^hi^ Tregs, were associated with transplant tolerance.

IL-10 (44), TGF-β1 (45), and CTLA4 (46) play important roles in Treg suppression of other immune cell populations. IFN-γ is a key cytokine in both acute T cell–mediated allograft rejection (47) and tolerance induction, where IFN-γ has been shown to be produced by allogeneic Foxp3^+^ Tregs (48). Therefore, we assessed expression of these genes in the spleen, ALN, DLN, and graft in tolerant and rejection groups by real-time RT-PCR. There were no differences in *Ctla4* and *Tgfb1* expression in all lymph organ types. However, significantly increased *Ifnγ* expression was observed in the DLN of tolerant mice at day 100 compared with naive mice, but *Il10* expression was significantly higher in the ALN but not the DLN of the tolerant mice at day 8 and day 100 after transplantation when compared with the naive mice (Supplemental Figure 10). At day 8, *Ctla4* and *Il10* gene expression was increased significantly in the grafts of tolerant mice when compared with rejection or control grafts. Meanwhile there were no differences in *Tgfb1* and *Ifnγ* gene expression between rejection and tolerant mice. At day 100, the expression of *Ctla4*, *Il10*, *Tgfb1*, and *Ifn*γ was significantly increased in tolerant grafts when compared with controls (Supplemental Figure 10).

The expression of *Il10*, *Tgfb*, *Ebi3* (reflecting IL-35) (49), and *Blimp1* (50) was assessed on sorted splenic CD127^hi^ Tregs of day 100 tolerant mice by real-time RT-PCR. Compared with naive Tregs, the expression of *Ebi3* was significantly increased in CD127^hi^ Tregs, but there was no significant difference in *Il10* gene expression between CD127^hi^ Tregs and naive Tregs (Figure 9D). EBI3 dimerizes with IL12p35 to produce IL-35, a regulatory cytokine secreted by Tregs and shown to have potent suppressive function in a variety of conditions, including cancer (51), autoimmunity (52), and infections (49). The expression of *Blimp1*, a marker of activated T cells and essential for the secretion of IL-10 by Tregs, was increased on CD127^hi^ Tregs compared with Tregs and Foxp3^–^ CD4^+^ T cells from naive mice (Figure 9D).

Taken together, this suggests that IFN-γ and the regulatory cytokines, particularly IL-35 and IL-10 from CD127^hi^ Tregs, play an important role in transplant tolerance.

## Discussion

Here, we show that Tregs are vital to maintaining islet transplant tolerance induced by CTL4-Fc/MR1 treatment. We further identify a subset of memory-like CD127^hi^ Tregs within the graft and lymphoid organs Tregs that is critical to maintenance of transplant tolerance. We demonstrated that Tregs differentiate to express high CD127, and increased CD127^hi^ Tregs were associated with strong activation of phosphorylation of STAT5 by IL-7. Under tolerizing conditions, Tregs encounter antigen in the DLN and migrate to the graft, where they encounter antigen in the context of the graft and as a result undergo further differentiation into memory-type Tregs with high CD127 and CD44 expression and coexpression of CD69 and CD103, identifying them as tissue Tregs. Functionally, these memory-like CD127^hi^ Tregs had potent suppressive function in very small numbers and had a transcriptional profile that matched those of tissue Tregs or tumor-associated Tregs.

In recent years, the importance of the differing functional subsets of Tregs has been recognized in several immune conditions (5, 10, 12, 32, 37, 49, 51, 53–55). Tissue Tregs perform important homeostatic and regenerative functions in multiple tissues, limiting the harmful effects of inflammation (6, 7, 32–37, 56, 57). Tregs are also abundant in many tumors, where their potent suppressive function inhibits antitumor responses (51, 58–69). There is a shared transcriptional trajectory between tissue Tregs found in different anatomical sites and across species (7, 33, 36, 56, 57). It has also been reported that precursors for ST2^+^ tissue Tregs undergo a stepwise reprogramming in secondary lymphoid organs driven by the transcription factor *Batf* (33). These same processes appear to be present in the transplant setting, following antigen priming and activation in the DLN, then migration to the graft, where they undergo further differentiation and are involved in graft homeostasis and suppression of the inflammatory response.

In the context of transplant tolerance, it is known, from experimental studies in mice, that donor-antigen-experienced Treg suppression leads to tolerance that is antigen specific and infectious (4, 12, 70). Therefore, T cell receptor affinity to antigen is an important component of their suppressive function, and continuous exposure to antigen is essential for their ongoing activation (71). However, it is not clear if these activated/effector Tregs partially differentiate into memory Tregs (4, 11, 12). However, by using the porcine NICC graft-tolerant model, relatively large numbers of indirectly activated Tregs can be isolated and phenotyped from specific compartments. In long-surviving tolerant mice, Tregs with a memory-like phenotype and CD127^hi^ Tregs were found in the spleen, DLN, and grafts. The transcriptional profiles of these memory-like CD127^hi^ Tregs had unique transcriptional and signaling features that distinguished them from classical lymphoid Tregs, as well as shared similarities with other tissue Treg phenotypes that have been described (6, 32–37, 55, 56, 58, 63, 72).

Expression studies in mice and humans suggest a large diversity of overlapping Treg subsets (73). Specialization allows the Tregs to match canonical features that define the T cell–mediated inflammatory response, such as CD4^+^ T cell–mediated Th1, Th2, Th17, and T follicular cell responses (74–76). Our studies show that both the lymphoid CD127^hi^ Treg subsets and/or graft Tregs found in the tolerized islet transplants express transcription regulators associated with B cells and immunoglobulin production, such as *Cd19*, *Cd79a*, *CD79b*, and immunoglobulin genes, in addition to the major survival factor of neutrophils *Serpinb1a* (Supplemental Table 2). As proposed by others, these data suggest that Tregs can adapt to the inflammatory environment and suppress the corresponding immune response using similar transcriptional drivers (77). These Treg findings reflect reports of tissue-resident memory CD8^+^ T cells that possess unique functional and transcriptomic signatures in different tissue environments (78–80).

Although this study has focused on the phenotype of CD127^hi^ Tregs and their capacity to suppress rejection and promote tolerance, the experimental model did rely on dual blockade of the B7/CD28 and CD40/CD154 pathways. Whether this is essential for the development of CD127^hi^ Tregs in transplant settings is uncertain. Clinically, belatacept is approved for use in transplantation, though early studies of CD154 blockade were ceased because of thromboembolic complications. However, newer agents blocking either CD154 or CD40 are undergoing clinical trials, and there is the potential, in the future, to assess the clinical value of dual costimulation blockade (81). Regardless of the induction agent, Tregs have been proposed as therapy for promoting transplant tolerance as well as suppressing autoimmune diseases, due to their unique suppressive function and stability. However, developing Tregs as a viable clinical therapy has had issues around selection, potency, and specificity; concerns regarding off-target effects; and limitations in suppressing established immune responses (82, 83). Further, our data suggest that strategies that promote or develop a tissue-specific, long-lived memory Treg may be more potent and effective in suppressing the cognate immune response and inflammation in a transplanted graft. In addition, after activation, Tregs may utilize IL-7 or IL-35 for survival, in preference to IL-2, which may act more broadly (10, 34, 36, 49, 51, 52).

In conclusion, this study demonstrates that memory-like CD127^hi^ Tregs develop in lymphoid organs and are further reprogrammed within the graft with an established phenotype as a memory-like CD127^hi^ tissue Treg with a unique molecular signature that is critical for maintaining tolerance. It provides a potential pathway for developing Tregs with this phenotype for potential clinical studies.

## Methods

### Sex as a biological variable.

Mice of both sexes were used in the study, and no significant difference was observed.

### Animals.

Breeding pairs of transgenic Foxp3^GFP^ mice expressing GFP under the control of the Foxp3 promoter (84) and DEREG mice (CD45.2), which carry the DT receptor and enhanced GFP transgene under the Foxp3 promoter (all on C57BL/6 background) (85), were provided by Alexander Rudensky (Sloan Kettering Institute, New York, New York, USA) and Tim Sparwasser (Institute of Medical Microbiology and Hygiene, Dresden, Germany), respectively. C57BL/6 Ly5.1 mice were purchased from the Animal Resource Center (Perth, Australia), and Ly5.1Foxp3^GFP^ mice (CD45.1) were bred at Westmead Animal Care Facility (Sydney, Australia). Rag^–/–^ mice were obtained from the Animal Resource Center (Perth, Australia) and Australia BioResources (Moss Vale, Australia). Newborn Westran pigs aged 2–7 days were obtained from either The University of Sydney Camden Campus or Bringelly Pig Farm.

### Mouse model of porcine NICC transplant tolerance.

Porcine NICCs were isolated from the pancreas of 1- to 3-day-old piglets and propagated in culture for 6 days as described previously (86) and in Supplemental Methods. DEREG mice were transplanted with 4,000 islet equivalent porcine NICCs under their renal capsules as described previously (17). The tolerant group of DEREG mice received an i.p. dose (500 μg/mouse) of MR-1 (Bioexpress) at days 0, 2, 4, and 6 and a single i.p. dose (500 μg/mouse) of CTLA4-Fc (NS-1, WEHI Antibody Facility, Australia) at day 0. The DEREG mice transplanted with porcine NICCs without treatment were the rejection group. The transfer of donor immune cells did not occur in this islet transplant model (Supplemental Figure 11). Graft function was assessed by histological examination and serum porcine C-peptide. Graft rejection was defined as no visible intact graft with no positive insulin staining and serum porcine C-peptide less than 10 pmol/L (17).

### Depletion of Tregs in DEREG recipients.

DEREG mice were used for specific depletion of Tregs (12). In addition to costimulatory blockade, the transplanted DEREG mice were administrated DT (Calbiochem) daily at 12 ng/g i.p. starting 3 days before transplantation and continued for 3 days at 8 ng/g i.p., followed by no injection for the next 3 days (depletion group). This was continued until day 17 after transplantation.

### Flow cytometric analysis and cell sorting.

Peripheral blood samples were collected from CTLA4-Fc/MR1–treated DEREG recipients with and without DT treatment, to assess the efficacy of depletion on days 0, 3, 10, and 15. Spleens, ALNs, DLNs, or grafts were collected on days 8 and/or ≥100 after transplantation from DEREG recipients with and without treatment of CTLA4-Fc/MR1 and control DEREG mice (without transplantation and no treatment) for assessment of T cell and Treg subsets. The peripheral blood samples in transplanted Rag^–/–^ mice were collected at day 72 after adoptive transfer of Tregs (day 49 after challenging Foxp3^–^ CD4^+^ T cells). Transferred Tregs from DEREG mice were identified by the expression of CD45.2, and Foxp3^–^ CD4^+^ T cells from Ly5.1Foxp3^GFP^ mice were identified by the expression of CD45.1. Flow cytometry analysis was performed on a BD LSRII and BD LSRFortessa. Data were acquired using BD FACSDiva Version 6 Software system, then analyzed using FlowJo V10 as described previously (87).

Cell sorting and purity (the percentage of selected cell subset) after sorting were performed on BD FACSAria II to attain a purity more than 95% for collected samples used for assessment of Treg function in Rag^–/–^ mice. The tolerant Tregs or memory-like CD127^hi^ Tregs were sorted from the spleens of DEREG recipients that received CTLA4-Fc/MR1 at least 100 days after transplantation. DEREG mice without transplantation were the source of naive Tregs. CD4^+^GFP^–^ T cells from Ly5.1Foxp3^GFP^ mice were sorted as Foxp3^–^ CD4^+^ T cells for rechallenge. Briefly, the CD4^+^ T cells were separated using the mouse CD4 (L3T4) microbeads (Miltenyi Biotec) according to the manufacturer’s instructions using the autoMACS Pro Separator (Miltenyi Biotec). These isolated CD4^+^ T cell samples were sorted to collect tolerant Tregs based on negative DAPI (Invitrogen) and positive expression of CD4 and GFP and memory-like CD127^hi^ Tregs further based on positive or negative expression of CD44, CD127, and CD62L (Supplemental Figure 9A).

Cell sorting was performed on BD Influx using a single-drop envelope to attain high purity (>98%) and accurate sort volumes for performing real-time RT-PCR and bulk RNA-Seq. The samples of spleen, DLN, and graft were collected from DEREG recipients with tolerized porcine NICC grafts at day 100 or DEREG mice without transplantation. Cell samples were stained and sorted for naive Tregs, CD4^+^GFP^–^ T cells based on negative DAPI and the expressions of CD4 and/or GFP, and CD127^hi^ Treg and CD127^–/lo^ Treg subsets further based on CD127 expression (Supplemental Figure 5). Splenic cells were depleted of CD4^+^ T cells using CD4 (L3T4) microbeads and stained and sorted for CD45^+^CD4^–^ based on expression of CD45 and negative expression of DAPI.

Staining protocols, including intracellular phosphorylated STAT5 staining, are described in detail in Supplemental Methods, and antibodies are listed in Supplemental Table 5.

### Response of Tregs to IL-7 and IL-2.

Cells of spleens, DLNs, and grafts from CTLA4-Fc/MR1–treated DEREG recipients day 100 after transplantation and naive mice were stimulated with murine IL-7 (PeproTech) or recombinant human IL-2 (Novartis) at a final concentration of 5 ng/mL or 320 ng/mL, respectively, for 5 minutes at 37°C in a water bath, then incubated for 25 minutes at 37°C with 5% CO_2_. PBS was used as control on spleen and DLN samples. After stimulation, cells were fixed with CytoFix (BD) and permeabilized with Phosflow Perm Buffer III (BD) before staining with CD4, CD127, and STAT5 antibodies. Flow cytometry analysis was performed on BD FACSymphony.

### Assessment of Treg function by adoptive transfer of Tregs and challenge with Foxp3^–^ CD4^+^ T cells in Rag^–/–^ mouse recipients of porcine NICC grafts.

Sorted memory-like CD127^hi^ Tregs were adoptively transferred intravenously into Rag^–/–^ mouse recipients of NICC xenografts on day 22 after transplantation. On day 45 after transplantation, these mice were challenged intravenously with CD4^+^GFP^–^ T cells at the ratio of 3:1 (CD4^+^ T/Treg). There were 5 transplanted groups, including Rag^–/–^ mice that were infused with naive Tregs, tolerant Tregs, and memory-like CD127^hi^ Tregs and were challenged with Foxp3^–^ CD4^+^ T cells. The 2 control groups included transplanted Rag^–/–^ mice without infusion of Tregs and Foxp3^–^ CD4^+^ T cells (transplant only, the positive islet control) and Rag^–/–^ mice transfused with CD4^+^GFP^–^ T cells (the negative islet control). All mice were sacrificed by ≥day 120 after transplantation (which was equal to post–adoptive transfer ≥100 days).

### Measurement of porcine C-peptide.

To assess graft function, the level of porcine C-peptide was measured in the serum of NICC graft recipients using Mercodia porcine C-peptide ELISA according to the manufacturer’s instructions.

### Histological examination of porcine NICC grafts.

Kidneys containing the grafts in DEREG recipients at days 8, 20, and ≥100 after transplantation and in Rag^–/–^ recipients day ≥122 after transplantation were fixed in 10% formalin and paraffin-embedded for H&E staining and insulin staining. Porcine endocrine cells were detected using immunohistochemistry by primary antibody polyclonal guinea pig anti-insulin (Agilent Dako) with secondary antibody rabbit anti–guinea pig IgG/HRP (Agilent Dako) on paraffin sections. Slides were incubated with DBA, stained with hematoxylin, and dehydrated as described previously (17). Slides were imaged on the NanoZoomer Slide Scanner using NDP.scan 2.2.60 software. Images were visualized using Aperio ImageScope (v.12.4.0.7018) software (Leica Biosystems).

Porcine endocrine cells also were identified using immunofluorescence staining with polyclonal guinea pig anti-insulin antibody (primary antibody) and with goat anti–guinea pig Texas Red (Abcam) as the secondary antibody on frozen OCT sections of grafts of rejection and tolerant mice (17). The slides were counterstained with DAPI Vectashield Mounting Medium (Vector Laboratories) (87). Slides were imaged on Olympus FV ≥1000 confocal microscope, with FV10-ASW 4.2 software.

Antibodies used are detailed in Supplemental Table 6. The staining protocols of immunohistochemistry and immunofluorescence are described in Supplemental Methods.

### Real-time RT-PCR.

RT-PCR was performed using TaqMan Gene Expression Assay (Thermo Fisher Scientific) according to the manufacturer’s instructions on the cDNA of spleen, ALN, DLN, and NICC graft with kidney capsule on day 8 and day 100 from DEREG recipients with and without CTLA4-Fc/MR1 treatment and control samples. Real-time RT-PCR was performed in duplicate using the Bio-Rad CFX96 in 96-well and 384-well plates (Bio-Rad). *Il10*, *Tgfb1*, *Ifnγ*, *Blimp1*, *Ctla4*, *Ebi3*, *Il12*, *Il7*, *Il18*, *Il33*, *Havcr2* (*Tim3*), *Hprt* (Thermo Fisher Scientific), and *Gapdh* (Applied Biosystems) (Supplemental Table 7). The mRNA expression was measured as the relative quantity of *Gapdh* or *Hprt* for normalized gene expression using the comparative Ct method.

### IMC.

All antibodies were validated, pretitrated, and supplied in per-test amounts by the Ramaciotti Facility for Human Systems Biology Mass Cytometry Reagent Bank, The University of Sydney, and listed in Supplemental Table 8. Reagent bank antibodies were purchased from Fluidigm in preconjugated form, or unlabeled antibodies were purchased and conjugated by the Ramaciotti Facility for Human Systems Biology with the indicated metal isotope using the MaxPAR conjugation kit (Fluidigm) according to the manufacturer’s protocol. Frozen OCT graft-kidney samples from DEREG recipients at days 8, 20, and 100 after transplantation were performed for IMC with staining protocol as in Supplemental Methods. Subsequent slides were stained for hematoxylin and insulin to confirm graft sites. IMC sections were acquired on a Helios time-of-flight mass cytometer coupled to Hyperion Imaging System (Fluidigm), using an Nd:YAG 213 nm laser (200 Hz, energy 4–8 dB). Laser ablation was performed at approximately 1 μm resolution. Slides were ablated in a semirandomized order over a period of 3 weeks, with machine calibration occurring after every shutdown. Ablated areas were those containing NICC graft sites, with the previously hematoxylin- and insulin-stained subsequent slides being used as guides. IMC data visualization, cell segmentation and region of interest extraction, and software for IMC analysis are described in detail in Supplemental Methods and listed in Supplemental Table 9.

### Bulk RNA-Seq library preparation, data visualization, and data analysis.

Amplified cDNA for each sample was generated directly from 1,000 sorted Tregs using the SMART-Seq v4 Ultra Low Input RNA Kit for sequencing (Takara Clontech) according to the manufacturer’s instructions. Barcoded cDNA libraries were generated with the Nextera XT DNA Library Preparation kit (Illumina) and sequenced with a 2 × 75 bp paired-end protocol with a v3 reagent kit on the MiSeq (samples of DLN) and NextSeq 500 instruments (Illumina) (samples of spleen and graft) according to the manufacturer’s instructions.

Bulk RNA-Seq library data were visualized. To evaluate gene expression patterns between models, we constructed an integrated gene expression object, read counts were imported into R, and background was removed (counts per million < 1 in all samples or not mapped specifically to gene features). ComBat-Seq from the R package Surrogate Variable Analysis (R package version 3.40.0.) (88) was used to generate batch-normalized counts. The resulting count matrix was converted into an edgeR DGEList object. Normalization was performed using the edgeR function calcNormFactors before MDS with the plotMDS function. The limma function voom was used to make weighted log-scaled expression values for heatmap visualizations using pheatmap (89) and to make candidate expression box plots using ggplot2 (90).

Library sequencing quality was determined using FastQC (Babraham Bioinformatics). Illumina adaptor sequence and low-quality read trimming (read pair removed if < 20 bp) was performed using Trim Galore (Babraham Bioinformatics). STAR (91) was used to align reads to mouse genome mm10 using Ensembl gene annotations as a guide. Read counts data corresponding to Ensembl gene annotations were generated using HTSeq (92). In this study, libraries were mapped to a mouse mm10 + EGFP genome with a 75%–85% unique mapping rate (Supplemental Table 10), consistent with low-input kits. All analyses were performed in the R Statistical Environment (R Core Team) with tidyverse (90). Briefly, counts data were background corrected and normalized for library size using edgeR (93). DEG were determined using the glmLRT (FDR < 0.05 as significance, or absolute log FC > 1.5 combined *P* < 0.01). DEG lists with absolute log FC > 1.5 combined *P* < 0.01 were functionally annotated with GO Biological Processes pathway analysis (Supplemental Table 3) (94).

### Statistics.

Statistical analysis was performed using GraphPad Prism 8.2. Differences between 2 groups were evaluated using 2-tailed unpaired *t* test, 2-tailed paired *t* test, or Mann-Whitney *U* test, while 3 or more groups were compared using a 1-way ANOVA followed by Tukeyʼs multiple-comparison test or Kruskal-Wallis test (nonparametric) followed by Dunnʼs multiple-comparison test. Data were expressed as mean ± SEM. *P* < 0.05 was considered statistically significant.

### Study approval.

Animal protocols were approved by the animal ethics committee of Western Sydney Local Health District, Westmead, New South Wales, Australia.

### Data availability.

All data presented in this manuscript are accessible in the Supporting Data Values XLS file. All RNA-Seq data have been deposited at National Center for Biotechnology Gene Expression Omnibus and are publicly available as of the date of publication (accession number GSE220447).

## Author contributions

YZ, LN, and HW as co–first authors contributed to performing experiments and establishing transplant models, analyzed data, and wrote the manuscript. YZ was responsible for the establishment of the transplant model and related experiments, and her name appears first because of her role in the initiation of the project; LN and HW were responsible for the phenotypic, transcriptomic, and functional experiments in vitro. YWQ contributed to performance of experiments and establishment of the transplant model and analyzed and visualized data. WJH performed islet transplantation and porcine NICC isolation and culture. AT, YVC, HB, and EJV contributed to performance of porcine NICC isolation and culture. GYZ and YMW contributed to establishment of Ly5.1Foxp3^GFP^ mice and discussion of transplant model establishment. BSG and JL contributed to performance of Bulk RNA-Seq and data analysis. NMR, GZ, and SY contributed to the discussion of the research data. SIA contributed to interpretation of this work and edited the manuscript. MH and PJO contributed to conception, design, and interpretation of this work and wrote and finalized the manuscript for publication.

## Supplementary Material

Supplemental data 1

Supplemental data 2

Supplemental table 10

Supporting data values

## Version 1 03/22/2024

Electronic publication
