# Supplementary material for: Intragraft memory-like CD127hiCD4+Foxp3+ Tregs maintain transplant tolerance
Source: JCI Insight. 2024 Mar 22;9(6):e169119. doi: 10.1172/jci.insight.169119 (PMC11063946; doi:10.1172/jci.insight.169119)
Supplement: Supplemental data 1 [file jciinsight-9-169119-s094.pdf]

Intragraft memory-like CD127^{hi}CD4^{+}Foxp3^{+} regulatory T cells maintain transplant tolerance

Yuanfei Zhao,[1] Leigh Nicholson,[1] Hannah Wang,[1] Yi Wen Qian,[1] Wayne J Hawthorne[1], Elvira Jimenez-Vera,[1] Brian S Gloss,[2] Joey Lai,[2] Adwin Thomas,[1] Yi Vee Chew,[1] Heather Burns,[1] Geoff Y Zhang,[3] Yuan Min Wang,[3] Natasha M Rogers,[1, 4, 5] Guoping Zheng,[1] Shounan Yi,[1] Stephen I Alexander,[3] Philip J. O'Connell,[1] and Min Hu[1, 5]

Supplemental Materials

Supplemental Methods

Supplemental Figure 1 to 11

Supplemental Table 1 to 9

Supplemental Methods

The isolation protocol for porcine neonatal islet cell-clusters and culture. Pancreas were extracted from two to seven days old piglets and chopped in 15ml of 1× Hanks Balanced Salt Solution (HBSS) without porcine sera into small pieces (about 2 mm in size) and then were digested with 1 mg/ml Collagenase Type V (Sigma–Aldrich) at 37° for 12-14 minutes and filtered through a metal mesh. Then the islets were washed twice with 1× HBSS solution with 1% heat inactivated porcine sera, and centrifuged at 300g for 1 minutes. The supernatant was aspirated, and the islets were cultured in 20ml Hyclone Ham's F-10 culture media (GE Healthcare) containing 10% porcine sera, 10 mM glucose, 2 mM L-glutamine, 100 U/ml penicillin, 100 µg/ml streptomycin, Hepes 80 mM, 10 mM nicotinamide, 50 mM isobutylmethlxanthine, CaCl2 0.236 g/l, and NaHCO3 21.3% at 37°C, 4.9% CO_2 in 150mm × 20mm Petri dishes. Media were changed on day 1 and 3, where the cells were settled, and the supernatant was removed. The supernatant was then spun down at 300×g for 1 minutes at room temperature, and the supernatant was again removed. The cell pellet was suspended in HAMS F-10 culture medium. On day 5, each dish was topped up by 10ml of HAMS F-10 culture media. Islets were collected on day 6-7 and their diameter measured and categorised under the microscope, with the smallest being 50µm, and increasing incrementally by 50µm to 400µm. To normalise islet volume, the islet equivalent (IEQ) was calculated from the diameters, assuming that 1 IEQ is equal to a spherical islet with a diameter of 150µm. The islets under each diameter category were then converted to IEQ and 4000 IEQ porcine-NICC for each transplant.

Single cell suspensions for flow cytometric analysis and cell sorting. For preparing single cell suspensions from PB, PB was collected from mice into 4% w/v sodium citrate solution (Sigma Aldrich), which was kept at 4ºC. The sample tubes were centrifuged at 300*g* for 10 minutes, and the supernatant was poured off. The samples were lysed using sterile Ammonium-Chloride-Potassium (ACK) lysis buffer (Lonza Bioscience) for 3 minutes at room temperature and centrifuged at 300×g for 10 minutes at 4°C, then were washed using the FACS buffer solution [containing Dulbecco's phosphate-buffered saline (DPBS),

0.5% bovine serum albumin (BSA) and 2mM EDTA)] twice at 4°C. The cells were resuspended in the FACS buffer solution at 1×10^6 cells/100 μl.

For preparing single cell preparation from spleen, the spleens were removed from mice and transferred to a tube with RPMI 1640 (Lonza) supplemented with 10% fetal calf serum (FCS), then dissociated in a gentle MACS C Tube (MiltenyiBiotec) using the gentle MACS Dissociator (MiltenyiBiotec) with 3ml of FACS buffer solution as per the manufacturer's instructions. The samples were centrifuged at 300×g for 30 seconds at 4°C, and were washed through 70μm cell strainer (BD Biosciences) with the FACS buffer solution and centrifuged again at 300×g for 10 minutes at 4°C, then incubated in ACK lysis buffer for 2 minutes at room temperature, and washed using the FACS buffer solution twice at 4°C. The cells were resuspended in the FACS buffer solution at 1×10^6 cells/100 μl.

For preparing single cell preparation from lymph nodes, the lymph nodes were removed from mice and transferred to a tube with 3ml RPMI 1640 medium containing 10%FCS and 2mM EDTA, the lymph nodes were gently ground through a 70μm cell strainer (BD Biosciences) in a petri dish into a single cell suspension, were transferred and centrifuged at 300×g for 10 minutes at 4°C in the FACS tube, and washed twice using the FACS buffer solution at 4°C. The cells were resuspended in the FACS buffer solution at 1 $\times 10^6$ cells/100 μl.

For preparing single cell preparation from graft infiltrating cells, the islet grafts were removed from mouse kidney and transferred to a petri dish with 3ml RPMI 1640 medium containing 10%FCS and 2mM EDTA, and were gently flushed with 19G needle syringe, then were ground gently through a 70μm cell strainer (BD Biosciences) into a single cell suspension. The samples were washed twice using the FACS buffer solution at 4°C and resuspended in the FACS buffer solution at 1×10^6 cells/100 μl.

Staining protocols for flow cytometric analysis and cell sorting. For the staining protocol to assess $CD4^+GFP^+$Tregs in PB samples, the single cell suspension samples in the FACS buffer solution at 1×10^6 cells/100 μl were blocked with 1μl of 1:100 dilution purified anti-mouse-CD16/CD32 (2.4G2, BD

Biosciences) for 20 minutes at 4ºC, then the samples were stained with 1μl of 1:100 dilution anti-mouse-CD4-Pacific blue for another 30 minutes at 4ºC. After washing twice in the FACS buffer solution at a speed of 300×g for 10 minutes at 4°C, samples were resuspended in the FACS buffer solution for analysis on a flow cytometry for GFP and CD4 expressions.

For the surface staining protocol of multicolor panels, fluorescent-antibody cocktails were made in the FACS buffer solution prior to staining, and for the panels that contain two BD Horizon Brilliant antibodies were pre-mixed in BD Horizon Brilliant Stain Buffer (BV buffer) (BD Biosciences) at a 1:2 ratio. The antibody concentration in the cocktails was based on antibody titration and product instructions. For Treg phenotyping panels, the samples of single cell suspension at 1×10^6 cells/100 μl were stained with viability dye Zombie yellow (BioLegend) for 30 minutes at 4ºC based on the manufacturer's instructions with a temperature modification, then washed with the FACS buffer solution and centrifuged at 300×g for 10 minutes at 4°C. The samples were resuspended in FACS buffer solution at 1×10^6 cells/100 μl, and were blocked with purified anti-mouse-CD16/CD32 for another 20 minutes at 4ºC, then stained with surface antibody cocktails for another 30 minutes at 4ºC. The samples were washed twice with the FACS buffer solution at a speed of 300×g for 10 mins at 4°C, and resuspended for acquisition.

For cell sorting, cell samples were stained with surfaces antibody cocktails after blocking with purified anti-mouse-CD16/CD32. 4',6-diamidino-2-phenylindole (DAPI) (Invitrogen) was used to stain samples after surface antibody staining.

For the Foxp3 intracellular staining protocol, the samples were prepared at 4ºC during processes according to the Foxp3/transcription Factor Staining Buffer Set protocol (eBioscienceTM). Briefly, after surface staining completion, the sample were fixed and permeabilized with transcription factor buffer set for 50 minutes at 4˚C, then washed with fixation washing buffer twice. Samples were stained with Foxp3 antibody for 40 minutes at 4˚C. Then the samples were washed twice with fixation washing buffer, and resuspended for acquisition.

For the phosphorylation of STAT5 intracellular staining, single-cell suspensions containing 2×10^6 cells from DLN and graft, and 10×10^6 cells from the spleen in 1 mL PBS/2% FCS were stimulated with murine IL-7 (5 ng/ml; PeproTech) or recombinant human IL-2 (320 ng/ml; Novartis) for 5 minutes at 37°C in a water bath, followed by 25 minutes in a 37°C incubator with 5% CO2. Samples were fixed with 1 mL CytoFix (BD Biosciences, warmed to 37°C before use) for 10 minutes at 37°C. These fixed cell samples were washed with PBS/2% FCS and then permeabilised with Phosflow Perm Buffer III (BD Biosciences) on ice for 30 minutes. Next, samples were washed with PBS/2% FCS, and stained with CD4, CD127, and STAT5 antibodies for 1.5 hours at room temperature in the dark. Finally, the samples were washed with PBS/2% FCS twice and then resuspended for acquisition.

Staining protocol of immunohistochemistry for insulin Staining. Formalin-fixed paraffin wax-embedded samples of kidney containing grafts were sectioned at 6-7μm. Slides were de-waxed in xylene and rehydrated in decreasing concentrations of ethanol, finishing in an H2O wash. Slides were incubated in 3% H2O2/methanol for 10 minutes at room temperature and washed in phosphate-buffered saline solution (PBS) with 0.05% Tween™ 20 (PBSt). Slides were blocked in PBSt with 7% rabbit serum for 20 minutes at room temperature and incubated in the primary antibody for 1 hour at room temperature. Slides were washed in PBSt and incubated in the secondary antibody mix for 30 minutes at room temperature. Slides were washed in PBSt, incubated in DAB for 3 minutes before rinsing in H2O. Slides were stained with hematoxylin and dehydrated in increasing concentrations of ethanol and xylene before coverslipping.

Staining protocol of immunofluorescence for insulin. Frozen OCT samples of kidney containing grafts were cryo-sectioned at 6-7μm. Antibodies used are detailed in table S5. Sections were fixed in 4% paraformaldehyde for 10 minutes at room temperature and washed in PBS solution. Slides were blocked in DPBS with 2% BSA for 20 minutes and then incubated in the primary antibody mix (diluted in 2% BSA/PBS) overnight at 4°C. Slides were then washed in PBSt and incubated in secondary antibody mix (diluted in 2% BSA/PBSt) for 1 hour at room temperature, in the dark. Slides were washed in PBSt and then counterstained with DAPI before being mounted and coverslipped.

Staining protocol of imaging mass cytometry. Frozen OCT NICC graft-kidney samples for imaging mass cytometry (IMC) were cryo-sectioned at 6-7μm two days before antibody incubation. Subsequent slides were stained for hematoxylin and insulin to confirm graft sites. Slide staining was split into two groups with evenly distributed samples from each experimental group to prevent both batch effects and inconsistent staining times. IMC slides were fixed with 100% methanol for 10 minutes at room temperature and washed in PBSt. Slides were blocked using 3% BSA/PBSt, with anti-CD16/32 added to block low-affinity Fc receptors. Slides were incubated with the primary antibody cocktail (Supplementary Table 6) overnight at 4°C. Slides were washed in PBSt and a secondary fixation of 4% paraformaldehyde was carried out at room temperature for 20 minutes. Slides were washed in PBSt and counter-stained with iridium-DNA-intercalator diluted in 3% BSA/PBSt for 30 minutes at room temperature. Slides were washed in PBSt with a final ultra-pure H2O 5 second wash before air-drying. Slides were stored in a sealed container at room temperature prior to acquisition.

IMC data visualisation, cell segmentation and ROI extraction. IMC data was visualised using MCD Viewer. Colour thresholding was performed on each channel to remove background noise and was consistently applied to the same channel across all samples. Channels of interest were overlayed and processed in ImageJ. Prior to quantitative analysis, cell masks for each sample were created using a process modified from the Bodenmiller Lab protocol by the Sydney Cytometry Facility. MCD files of each sample acquired from the Hyperion™ Imaging System were extracted to produce tiff image files corresponding to each marker channel. CellProfiler was then used to create random cropped sections for each sample. These crops were used in Ilastik to train feature identification using supervised machine learning. Pixels were classified as belonging to either nucleus, cytoplasm, or background, generating a probability map. The probability map was then imported back into CellProfiler to generate an individual cell mask file for each sample, identifying cell nuclei and cytoplasmic regions, for use in all proceeding analysis. A false-positive edge artifact in the B220_176Yb channel was observed in all 24 samples, resulting in a strong signal running the length of the tissue edge, which has commonly been reported in similar methods. Since this artefact was

consistent across every sample, an additional step was incorporated to exclude the edge-regions using a gating method common to IMC ROI extraction. IMC data for each sample, including respective cell masks, was loaded into computational histology topography cytometry analysis toolbox (histoCAT). Each sample was then manually gated to exclude the edge-region (distance) and all single-cell information (marker abundance, xy spatial data and neighbourhood data) was extracted as an csv file per sample.

Archsinh transformed expression values were scaled per image and cells were assigned as positive for each marker based on scaled values greater than background and verified by manual image analysis. Cell types were assigned according to standard type marker designation. Heatmaps were generated using the SPECTRE do.aggregate function (95) and pheatmap (89). Other visualizations were generated using ggplot2 (90).

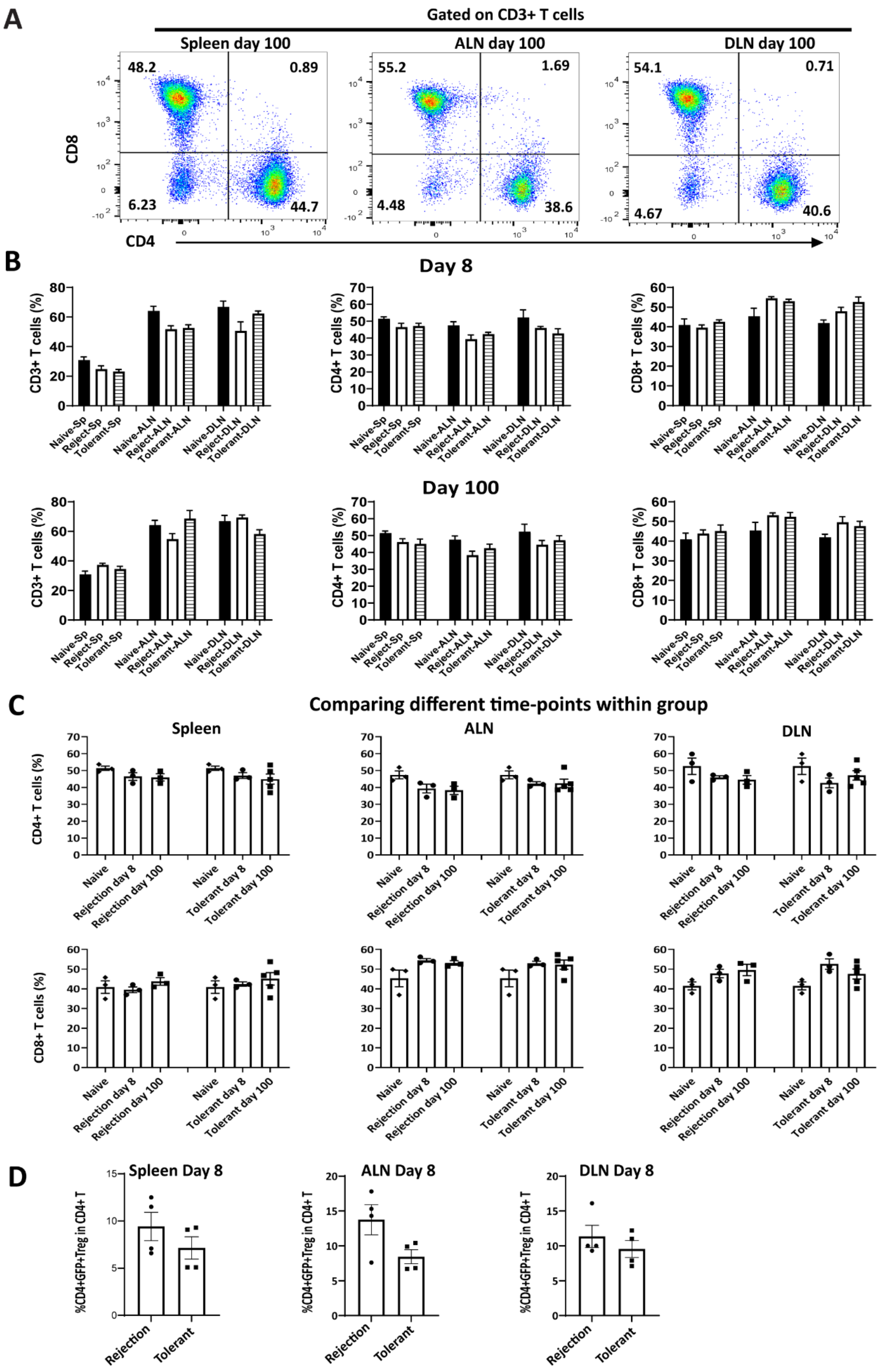

Supplemental Figure 1. T cells in spleen and lymph nodes of DEREG-mouse recipients receiving porcine-NICC transplantation. (A) Representative pseudocolor plots of CD4 versus CD8 (gated on

$CD3^+$T cells) in spleen, auxiliary lymph node (ALN) and graft-draining LN (DLN) in tolerant-group at day-100 post-transplantation. **(B)** Proportion of $CD3^+$ T cells in the spleen, ALN and DLN; and proportions of $CD4^+$ and $CD8^+$ T cells within total $CD3^+$T cells in the spleen, ALN and DLN from DEREG-mice without transplant and no treatment (naïve-Sp, naïve-ALN, naïve-DLN) (naïve-group, *n* = 3 - 6); and from DEREG-mouse recipients receiving porcine-NICC transplants and CTLA4-Fc/MR-1 treatment (tolerant-Sp, tolerant-ALN, tolerant-DLN) (tolerant-group, *n* = 3 - 6) and no treatment (reject-Sp, reject-ALN, reject-DLN) (rejection-group, *n* = 3) on day-8 and day-100 post-transplantation. **(C)** The comparisons of $CD3^+CD4^+$T and $CD3^+CD8^+$T cells as a proportion of $CD3^+$T cells in the spleen, ALN and DLN within tolerant-group or rejection-group respectively. **(D)** Proportion of $CD4^+GFP^+$/$Foxp3^+$Tregs within $CD4^+$T cells of spleen, ALN and DLN at day-8 post-transplantation. Flow cytometry analysis was performed. Kruskal-Wallis test was used in *(B)* and *(C)*. An unpaired t test (2-tailed) was used in *(D)*. Error bars indicate the mean ± SEM.

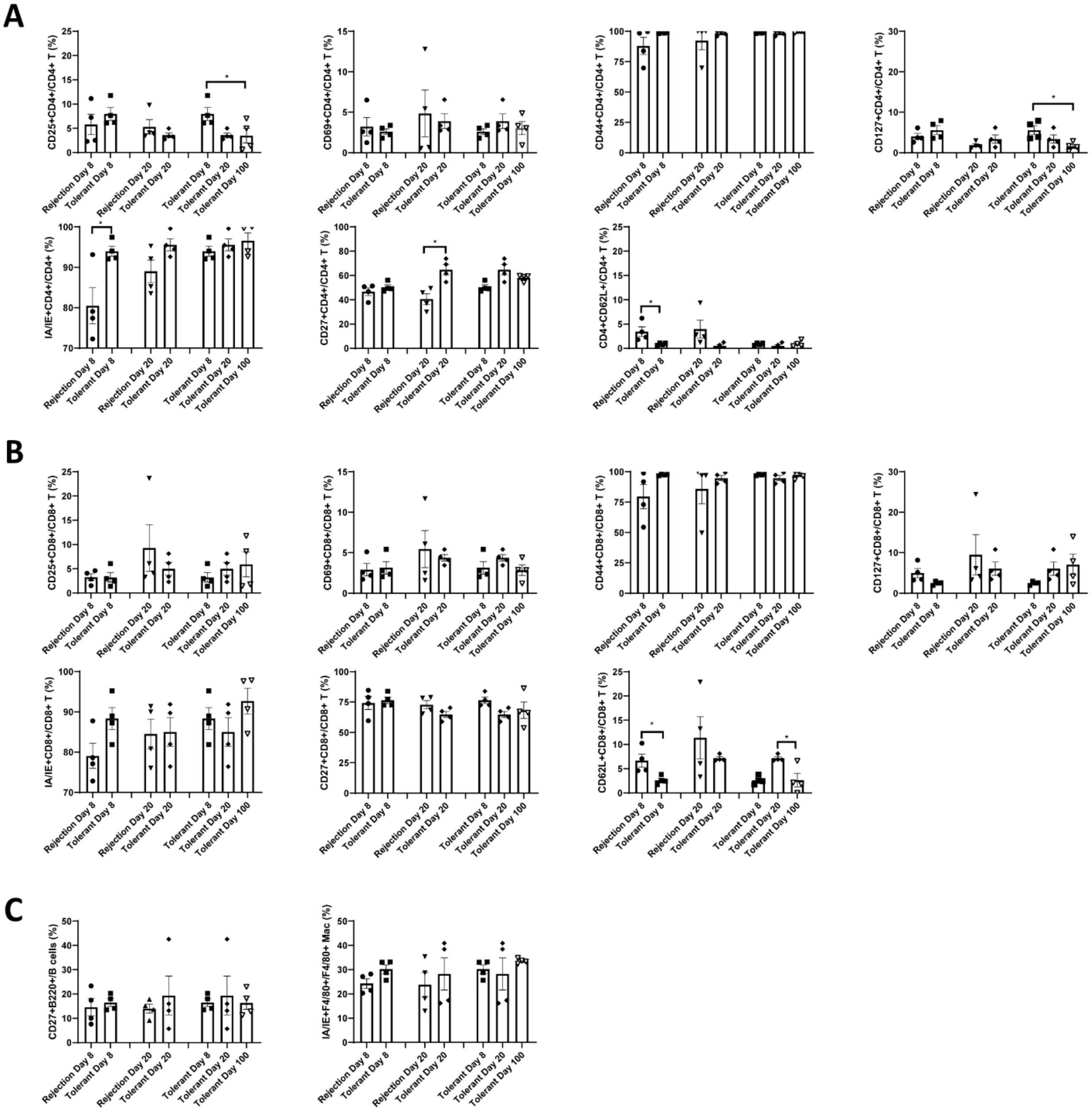

Supplemental Figure 2. The status of immune cell subsets in the graft site by imaging mass cytometry. Expressions of CD25, CD69, CD44, CD127, IA/IE, CD27, CD62L as the proportion (%) in (**A**) CD4^{+}T cells (CD45^{+}CD3^{+}CD4^{+}CD8^{-}Foxp3^{-}) and (**B**) CD8^{+}T cells (CD45^{+}CD3^{+}CD8^{+}CD4^{-}); (**c**) and expression of CD27 or IA/IE as the proportions (%) in B cells (CD45^{+}CD3^{-}B220^{+}) or macrophages (CD45^{+}F4/80^{+}) respectively were shown in tolerant-group (day-8, 20, 100) and rejection-groups (day-8, 20). An unpaired t test was used for comparing rejection-group and tolerant-group (except CD62^{+}CD4^{+} T cells used the Mann-Whitney test) and a one-way ANOVA followed was used for comparing day-8, day-20, and day-100 within tolerant-group (except CD62^{+}CD4^{+} and CD62^{+} CD8^{+} T cells used Kruskal-Wallis test). Label of statistical significance: *P <0.05. Error bars indicate the mean ± SEM.

A

Laser (nm)	355		403			488	561	639	
Laser (mw)	20		50			50	50	40	
Band pass filter	379/28	740/35	440/40	580/50	710/50	530/30	586/15	670/14	780/60
Panel 1	CD4 (BUV395)	CD3 (BUV737)	CD44 (BV421)	Zombie Yellow	MHCII (BV711)	Foxp3 (GFP)	CD39 (PE)	CD25 (APC)	CD127 (APC-Cy7)
Panel 2	CD4 (BUV395)	CD3 (BUV737)		Zombie Yellow	MHCII (BV711)	Foxp3 (GFP)	CD62L (PE)	CD27 (APC)	

B

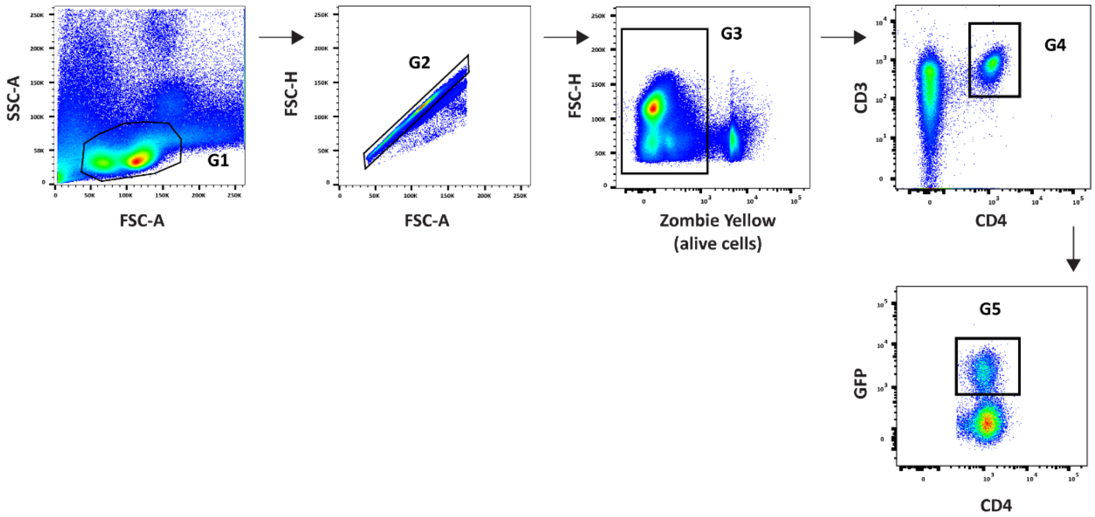

Supplemental Figure 3. Multicolour flow cytometry panels for phenotyping Tregs. (**A**) The two multicolour antibody panels. Two Treg panels were used for flow cytometry analysis containing 9 parameters in the panel 1 and 7 parameters in panel 2 acquiring on BD-LSR Fortessa. (**B**) Gating strategies for phenotyping Tregs. The first gate (G1) was for lymphocytes using FSC-A versus (vs) SSC-A. The second gate (G2) was to exclude double cells using FSC-A vs FSC-H. The third gate (G3) was to include only live cells using Zombie Yellow vs FSC-H. The fourth gate (G4) was on $CD3^+CD4^+$T cells (for Figure 4 and Supplemental Figure 5A), and the fifth gate (G5) was on $CD4^+GFP^+/Foxp3^+$ Tregs (for Supplemental Figure 5B).

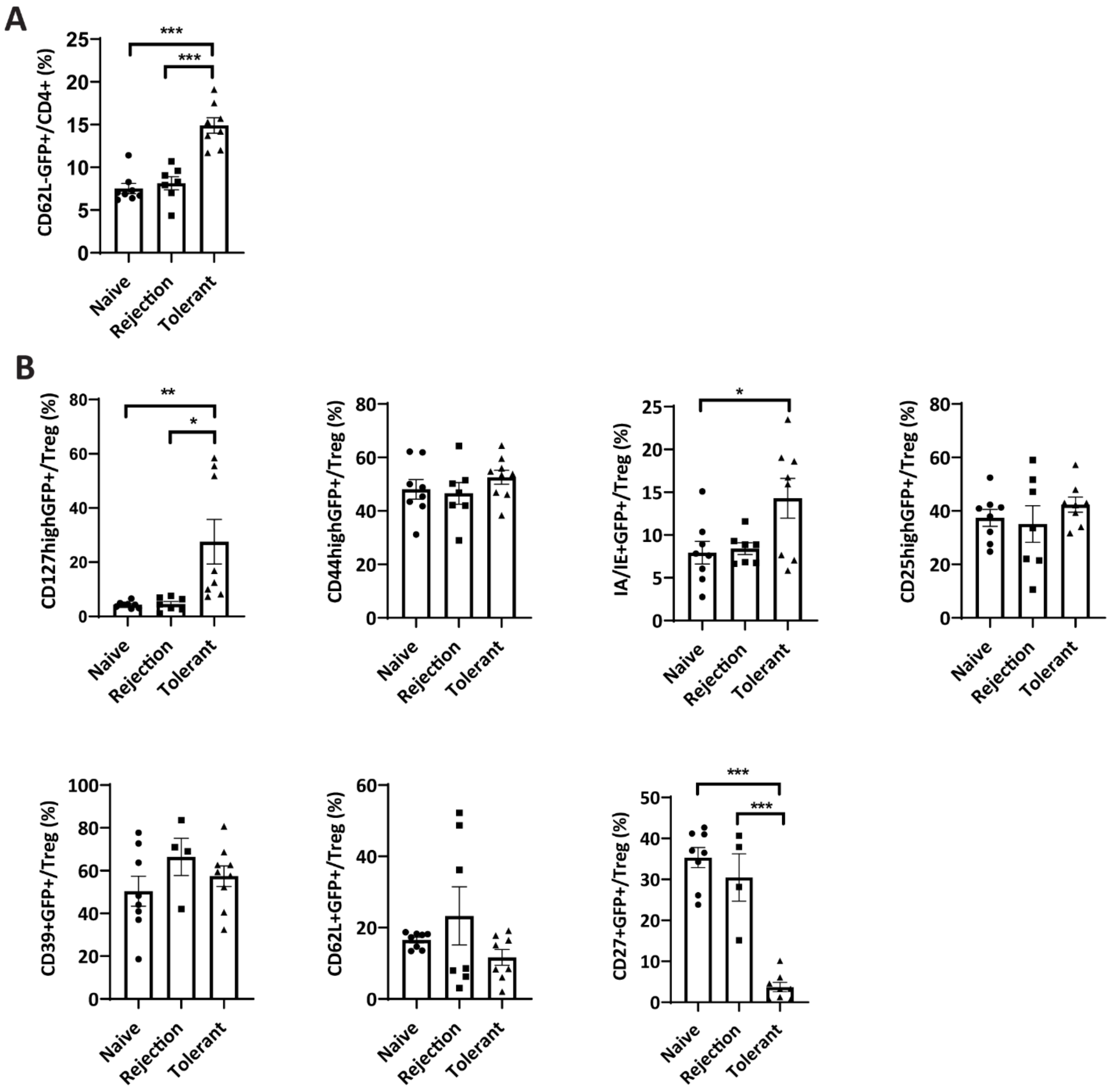

Supplemental Figure 4. Immune phenotype of Tregs in the spleens in naïve-, tolerant- and rejection-groups. (**A**) Proportions of CD62L$^-$GFP$^+$Tregs in CD4$^+$T cells, and (**B**) proportions of CD127^{high}GFP$^+$, CD44^{high}GFP$^+$, IA/IE$^+$(MHC-II)GFP$^+$, CD25^{high}GFP$^+$, CD39$^+$GFP$^+$, CD62L$^+$GFP$^+$, CD27$^+$GFP$^+$Tregs within CD4$^+$GFP$^+$/Foxp3$^+$ of spleens in the rejection-group (n = 4 -7) and the tolerant-group (n = 8 - 9) on day-100 post-transplantation, and naïve-group (n = 8) are shown. Flow cytometry analysis was performed. A one-way ANOVA followed by Tukey's multiple comparison was used. Data were from three independent experiments. Label of statistical significance: *P <0.05, **P <0.01, ***P < 0.001. Error bars indicate the mean ± SEM.

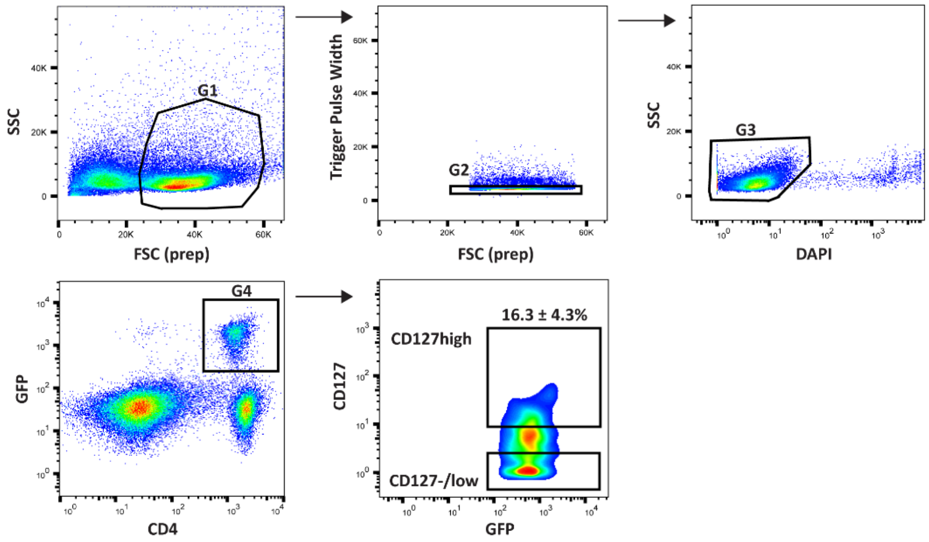

Supplemental Figure 5. The gating strategies for sorting CD127^{high} and CD127$^{-/low}$ Tregs and the proportion of CD127^{high}Tregs in DLN of tolerant-mouse recipients. The first gate (G1) on FSC (prep) vs SSC for lymphocytes, the second gate (G2) on FSC (prep) vs Trigger Pulse width for single cell staining, the third gate (G3) on DAPI vs SSC to exclude dead cells, the fourth gate (G4) on CD4 vs GFP for separating Foxp3^{+}Tregs, GFP vs CD127 for sorting CD127^{high}Tregs and CD127$^{-/low}$Tregs. Representative pseudocolor-plot was from DLN samples on day-100 post-transplantation from DEREG recipients receiving CTLA4-Fc/MR-1 treatment (n = 6) (CD127^{high}Tregs: 16.3 ± 4.3%). Cell sorting was performed on BD-Influx (BD Biosciences).

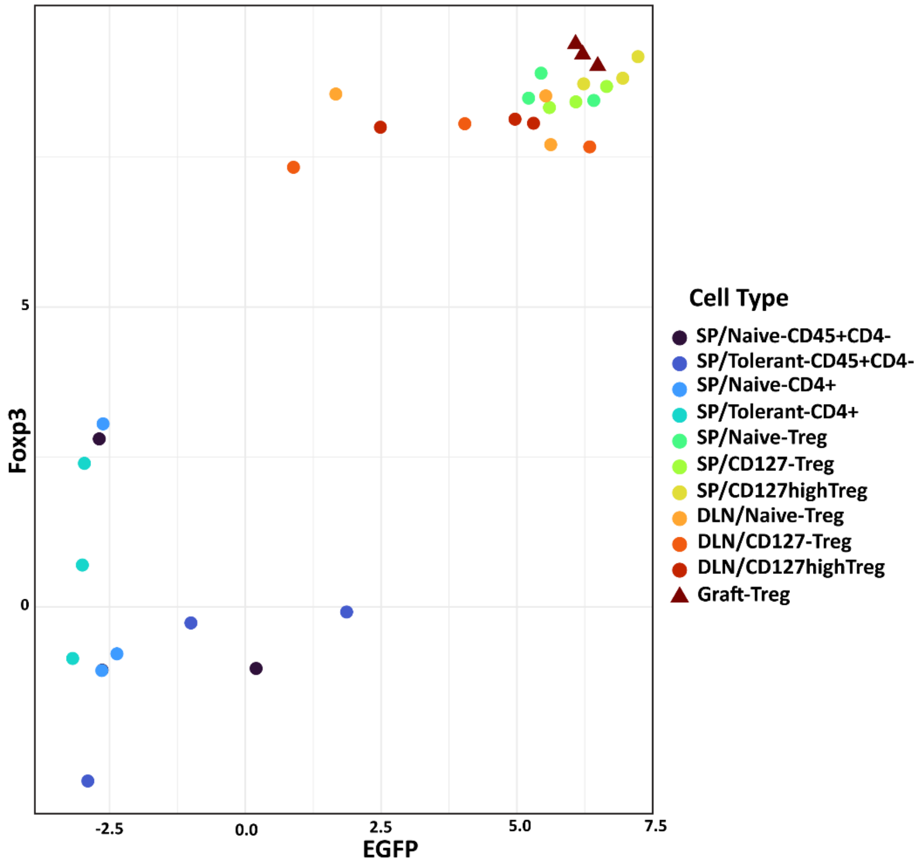

Supplemental Figure 6. The relation between Foxp3 gene and GFP gene expressions. Dot plot of Foxp3 vs GFP genes showing the segregation of Tregs and non-Foxp3 immune cell subsets by Bulk RNA-seq data analysis. Treg subsets included $CD127^{high}CD4^{+}GFP^{+}$Treg and $CD127^{-/low}CD4^{+}GFP^{+}$Treg subsets of spleen (SP/CD127highTreg & SP/CD127-Treg) and DLN (DLN/CD127highTreg & DLN/CD127-Treg), and graft infiltrating $CD4^{+}GFP^{+}$Tregs (graft-Tregs) from DEREG-recipients in tolerant-group day-100 post transplantation; and $CD4^{+}GFP^{+}$Tregs from spleen (SP/Naive-Tregs) and DLN of DEREG naïve-mice (DLN/Naive-Tregs). Non-Foxp3 subsets included $CD4^{+}GFP^{-}$T cells and $CD45^{+}CD4^{-}$ immune cells from spleens of DEREG naïve-mice (SP/Naïve-CD4+ and SP/Naïve-CD45+CD4-) and spleens of tolerant-group DEREG-recipients (SP/Tolerant-CD4+ and SP/Tolerant-CD45+CD4-).

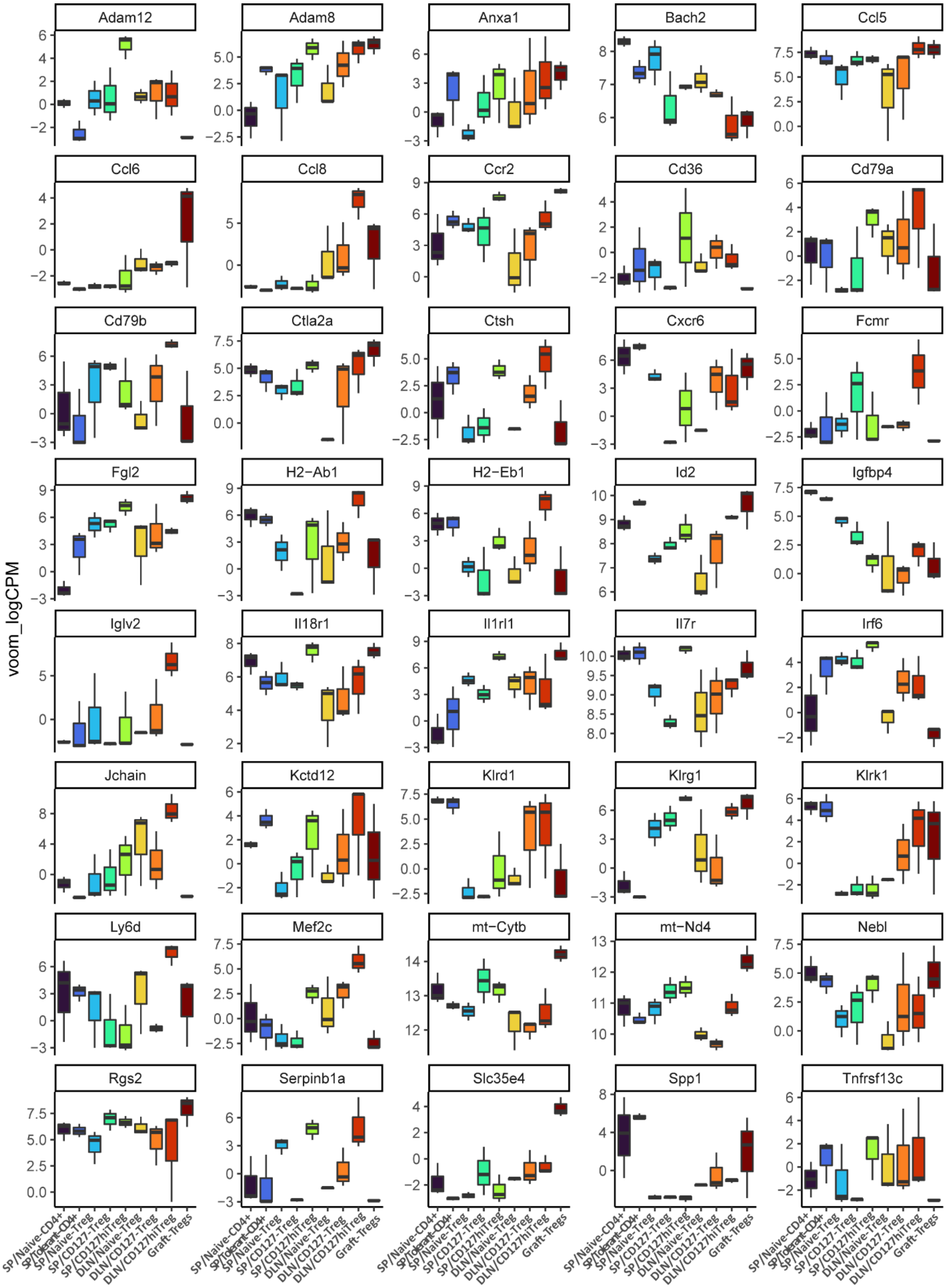

Supplemental Figure 7. Boxplots of selected 40 DEGs. Selected 39 DEGs with significant difference (FDR < 0.05) on SP/CD127high(hi)Tregs, DLN/CD127hiTregs, or graft-Tregs compared to naïve-Treg, or CD127-Treg subsets; and the enhanced DEG of *Klrg1* (FDR < 0.05) on SP/CD127hiTregs, DLN/CD127hiTregs, and graft-Tregs compared to splenic CD4^{+}T cells (tendency of enhanced *Klrg1* gene expression on SP/CD127hiTregs, DLN/CD127hiTregs, and graft-Tregs compared to naïve-Treg or CD127-Treg subsets).

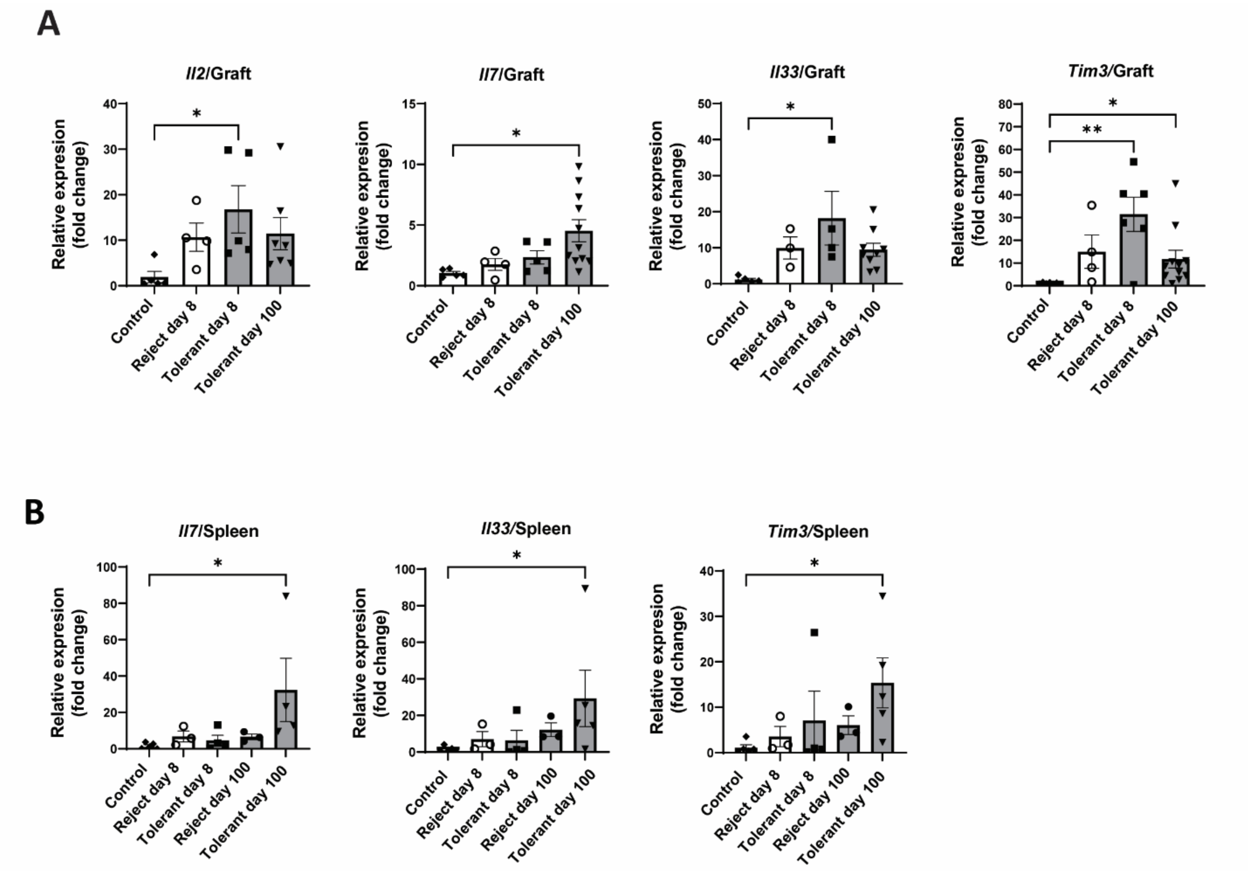

Supplemental Figure 8. Expressions of *Il12, Il17, Il33, Tim3* in DEREG-mouse recipients receiving porcine-NICC grafts. (**A**) Real-time RT-PCR measurement of *Il2, Il7, Il33 and Tim3* expressions of grafts and **(B)** spleens from mouse-recipients receiving porcine-NICC transplantation in rejection-group (day-8, n = 3 - 4), tolerant-group (day-8, n = 4-5 and day-100, n = 4 - 11) and control sample (n = 5) (kidney capture of naïve-mice with porcine-NICCs). Kruskal-Wallis test was used for the comparison of *Il7, Il33* and *Tim3* expressions in spleen and *Il2, Il33* and *Tim3* expressions in grafts between groups. A one-way ANOVA was used for the comparison of *Il7* expression in grafts between groups. Data were from three independent transplant experiments and shown as mean ± SEM. *P <0.05, **P < 0.01.

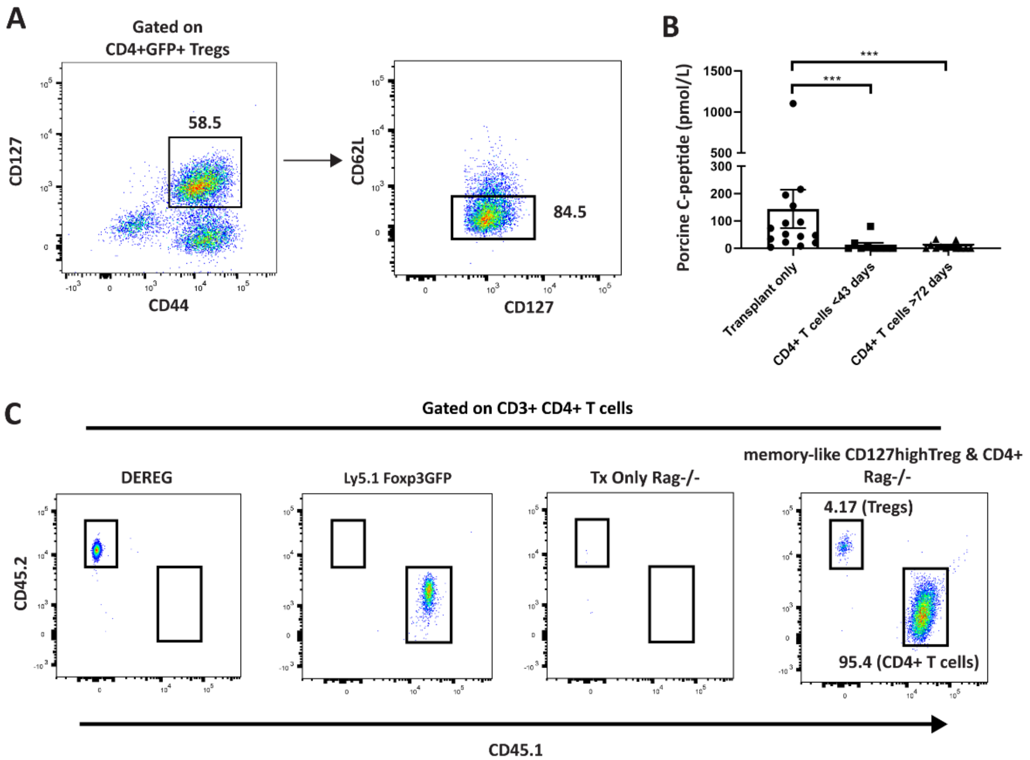

Supplemental Figure 9. Adoptive transfer of Tregs and challenge of CD4$^+$T cells in Rag$^{-/-}$ mice receiving porcine-NICC transplantation. (**A**) Sorting memory-like CD127^{high}Tregs from DEREG-mouse recipients in tolerant-group ≥100 days. After gating on CD4$^+$GFP$^+$/Foxp3$^+$Tregs (similar as Treg phenotyping gating steps in Supplementary Figure 4), then gating on CD44$^+$CD127^{high}Tregs, and collected CD127^{high}CD62L$^-$Tregs. Cell sorting was performed on BD-AriaII. (**B**) NICC-graft function by porcine C-peptide. The serum porcine C-peptide in Rag$^{-/-}$-mice recipients of NICC grafts without cell infusion (Transplant only) (n = 13 mice with 15 samples including one at day-29, 3 samples at day-71, and 11 samples ≥122 post-transplantation), and in Rag$^{-/-}$-mice recipients of NICC grafts that were only challenged with nonFoxp3CD4$^+$GFP$^-$T cells (the dose range from 6×10^4 to 2×10^6) at day from 29 to 42 days after cell challenge (CD4$^+$T cells < 43 days) (n = 9 mice including 3 mice with 1×10^5, 5×10^5, and 2×10^6 CD4$^+$T cells respectively at day-29 after challenge; 2 mice with 3.3×10^5 CD4$^+$T cells at day-35 after challenge; and 4 mice with 6×10^4, 2×10^5, and 6×10^5, and 2×10^6 CD4$^+$T cells at day-42 after challenge respectively); and > 72 days after challenge of CD4$^+$T cells (CD4$^+$T cells > 72 days) (n = 12 mice, dose range from 6×10^4 to 6×10^5, the number of mice in each dose listed in Supplementary Table 1). **(C)** Tregs and non-Foxp3 CD4$^+$T cells both existed in transplanted Rag-/- mice on 72 days after adoptive transfer of the Tregs that was 49 days after the challenge of CD4$^+$GFP$^-$T cells (Treg: CD4$^+$T cells = 1: 3). The representative pseudocolor plots of CD45.1 vs CD45.2 (gated on CD3$^+$CD4$^+$T cells) showed the CD45.1$^+$ and CD45.2$^+$ cells in PMBCs of DEREG naïve-mice (CD45.2) and Ly5.1Foxp3GFP (CD45.1) naïve-mice; Rag$^{-/-}$-mice receiving porcine-NICC transplantation (Tx) only, and Rag$^{-/-}$-mice adoptive transferred with memory-like CD127^{high}Tregs (2.8×10^5)(CD45.2) and challenged with nonFoxp3CD4$^+$GFP$^-$T cells (8.4×10^5)(CD45.1) on day-72 after adoptive transfer of Tregs i.e 49 days after challenge with CD4$^+$T cells (memory-like CD127^{high}Treg and CD4+/Rag-/-). Kruskal-Wallis test followed by Dunn multiple comparison test was used to compare the levels of serum porcine C-peptide. ***P < 0.001. Error bars indicate the mean of SEM.

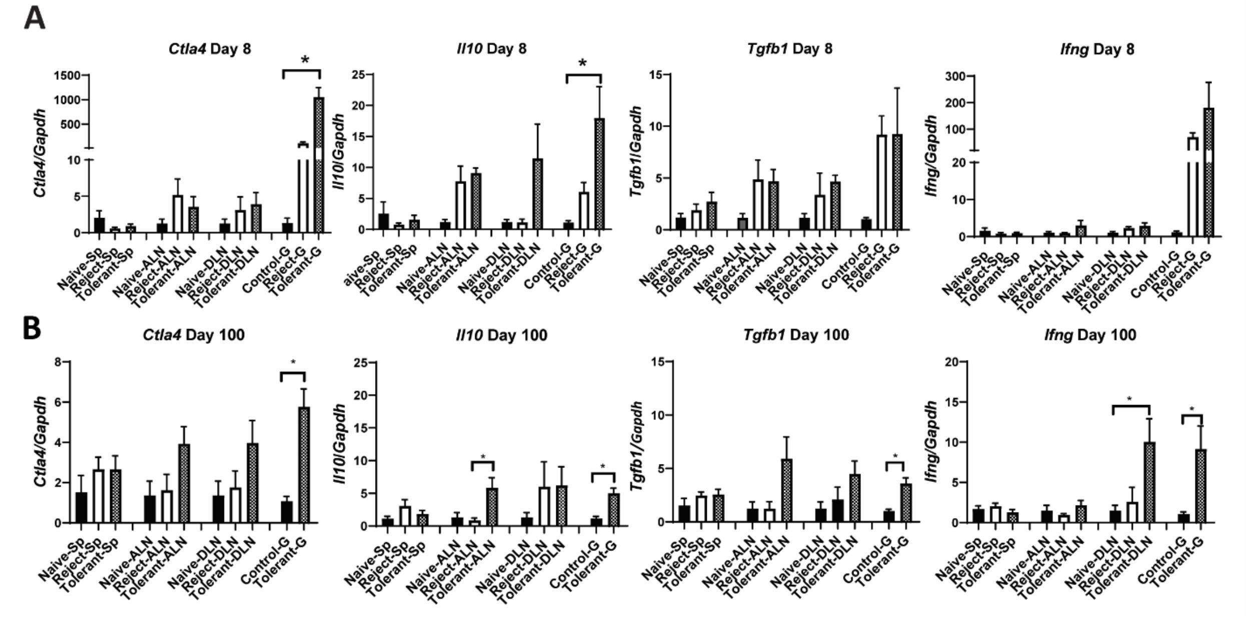

Supplemental Figure 10. Expressions of *Ctla4, Il10, Tgfb1* and *Ifng* on DEREG-mouse recipients receiving porcine-NICC transplantation. (**A**) Expression of *Ctla4, Il10*, *Tgfb1* and *Ifng* in the spleens, ALNs, DLNs and porcine-NICC-grafts of tolerant-group (Tolerant-Sp/ALN/DLN/G) (*n* = 3 - 6) and rejection-group (Reject/-Sp/ALN/DLN/G) (*n* = 3 - 5) at day-8 **(B)** and /or day ≥100 post-transplantation were assessed by real-time RT-PCR analysis using TaqMan® gene expression assay. The control samples included spleen (Naïve-Sp), ALNs (Naïve-ALN), and DLN (Naïve-DLN) from the naïve-mice group (*n* = 3), and control graft-sample (Control-G)(kidney capture of naïve-mice with porcine-NICCs) (*n* = 3). Kruskal-Wallis test was used for the comparison of three groups, and unpaired the Mann-Whitney test were used for the comparison of two groups. Label of statistical significance: **P* <0.05. Error bars indicate the mean ± SEM.

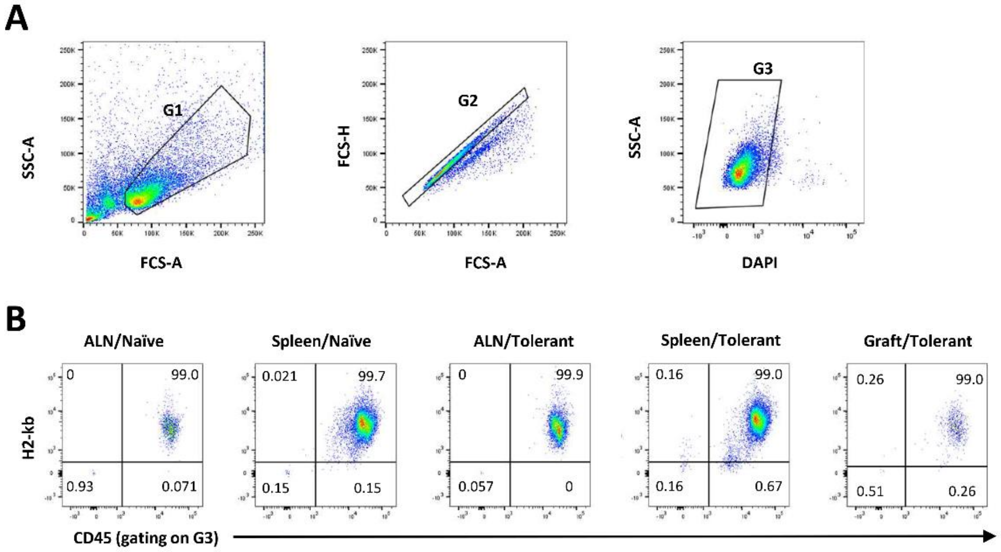

Supplemental Figure 11. CD45$^+$ immune cells were from mouse-recipient in transplant tolerance. (A) Gating strategies. The first gate (G1) on FSC-A vs SSC-A to exclude debris, the second gate (G2) was to exclude double cells using FSC-A vs FSC-H, and the third gate (G3) on DAPI negative to exclude dead cells. **(B)** The pseudocolor-plots of CD45 versus H2-Kb (mouse MHC class 1 for C57BL6 background) (gated on DAPI negative cells, the G3), showed CD45$^+$ cells in ALN, spleen and grafts tolerant-mouse were from DEREG-recipient (C57BL6 background). There was no difference of the proportion of CD45$^+$H2-Kb$^+$ cells in DEREG-recipient and DEREG mouse without transplantation (naive-group).

Supplemental Table 1. The number of DEGs from 15 paired cross-comparisons

**Pairwise comparison**	**[†]DEGs all**	**DEGs upregulated**	**DEGs downregulated**
SP/Tolerant-CD45+ vs[‡] SP/Naïve-CD45+	6	2	4
SP/Tolerant-CD4+ vs SP/Naïve-CD4+	20	16	4
SP/Naïve-CD4+ vs graft-Treg	469	253	216
[§]SP/CD127highTreg vs Graft-Treg	129	101	28
[§]SP/CD127-Treg vs Graft-Treg	104	77	27
[§]Graft-Treg vs SP/Naïve-Treg	129	29	100
[§]SP/CD127highTreg vs SP/CD127-Treg	30	19	11
SP/Naïve-CD4+ vs spCD127highTreg	362	142	220
[§]SP/CD127-Treg vs SP/Naïve-Treg	5	0	5
SP/Naïve-CD4+ vs SP/CD127-Treg	290	111	179
[§]SP/CD127highTreg vs SP/Naïve-Treg	14	7	7
SP/Naïve-CD4+ vs SP/Naïve-Treg	166	50	116
[§]DLN/CD127highTreg vs DLN/Naïve-Treg	9	7	2
[§]DLN/CD127-Treg vs DLN/Naïve-Treg	0	0	0
[§]DLN/CD127highTreg vs DLN/CD127-Treg	7	7	0

[†]DEGs: differentially expressed genes with false discovery rate < 0.05. Total 1740 DEGs.

[‡] vs: versus

[§]9 paired cross-comparisons within 7 Treg subsets.

Supplemental Table 2. Selected DEGs in lymphoid CD127^{high}Tregs and graft-Tregs and their functions

[†]Up DEGs	[‡]Treg subsets	Expression of the gene/encoded protein in tissue/tumor-Tregs or association in other studies	Function or association in other studies
Adam8	[§]I/II/III	Intestine tissue-Treg (96)[¶](ht), Tumor-Treg (63) [#](mt)	Associated with activated Tregs in chronic helminth infection (97)
Anxa1	I/II/III	Triple-negative breast cancer -Treg (59) (ht)	Enhanced suppressive function of tumor-Treg (59)
Ccl5	I/II/III	Pancreatic cancer-Treg (mt) (65)	Associated with Treg migration and homing into tissue (65)
Ccr2	I/II/III	Tumor-Tregs (58), and adipose (6, 36, 55), muscle (32) and intestine (34) tissue-Tregs (mt); adipose tissue-Treg (36)(ht)	Migration of Treg into inflamed tissues and migration of tissue-Treg from tissues to DLN (98).
Ctla2a	I/II/III	Tissue-Treg (63) (ht)	Associated with activated Tregs in chronic helminth infection (97).
H2.Ab1<br>*H2.Eb1*<br>*(HHC-II)*	I/II/III<br>I/II	**HLA-DR on effector-Tregs (99), and uterine-Tregs during pregnancy (100) (ht)	Associated with cell contact-dependent suppression [††](54)
Id2	I/II/III	Adipose tissue-Tregs (38) (mt)	Critical for tissue-Treg differentiation, survival, and function (38)
Il7r	I/II/III	Skin tissue-Tregs (10) (mt)	Associated with skin memory tissue-Treg survival supported by IL-7 (10)
Kctd12	I/II/III	Intestinal tissue-Tregs (101) (mt)	Associated with intestinal tissue-Tregs for the establishment of host–microbe symbiosis (101).
[‡‡]*Klrg1*<br>*Il1rl* (encodes ST2)	I/II/III<br>I/III	Adipose(6, 33, 36), skin, colon, lung (33), intestine(34), brain (35), muscle (32), and [§§]AKI kidney tissue-Tregs (37) (mt); adipose-Tregs (36) (ht)	*Klrg1:* maturation marker, important in immune regulation within tissue sites promoting Treg homeostasis (102).<br>*Il1rl:* associated with tissue-Treg homeostasis and tissue repair in response to alarmin IL-33 (34, 36) .
Fgl2	I/III	Tumor-Tregs (69) (mt and ht)	Activating Tregs (29, 31, 103).

Il18r1	I/III	Lung tissue-Tregs (72)	Associated with tissue-Treg homeostasis and tissue repair in response to IL-18 (72)
Ifngr1	I/III	Tolerant allograft-Tregs (12) and donor reactive Tregs in transplant tolerance (48) (mt)	Critical for long-term allograft survival induced by blocking T cell co-stimulation pathways (104), and maintaining donor reactive Treg survival in transplant tolerance (105)
Nebl	I/III	Muscle tissue-Tregs (32) (mt)	Contractile muscle function (106)
Rgs2	I/III	Kidney tissue-Tregs in later time of AKI (37), skin and colon (7) (mt)	Maybe associated with Treg migration (107)
Ccl8	II/III	Fibrosarcomas tumor-Tregs (67) (mt)	Maybe associated with migration of Tregs into tumour (108)
Cxcr6	II/III	Colon tissue-Tregs (7) (mt & ht), kidney cancer-Treg (66) (ht)	Treg tissue-specific migration and homing (66)
Klrk1	II/III	Liver cancer-Tregs *(60)*(mt)	Maybe associated with Treg suppressive function *(60)*
Ly6d	II/III	Tumor microenvironment of colorectal cancer in patients (109) and lung cancer in mice (110) with increased Tregs	Maybe associated with Treg migration into tumor (109)
Plac8	II/III	Expression on $CD39^-$Tregs, but not $CD39^+$Tregs (111) (ht)	Unclear Treg function.
Cd19<br>*Igkv8-30*<br>¶¶Ig/gene[a]<br>*Cd79a*<br>##Ig/gene[b]<br>*Cd79b*	II/III<br>II/III<br>I/II<br>I/II<br>II<br>II	*Igkv4-1, Igll1, and Igkv1d-13* expression in PB, and *Cd20, Foxp3* in urine of tolerant kidney transplant patients (112), *Cd79b* expression in uterine-Tregs during pregnancy (100) (ht)	The function of B cell related genes in $Foxp3^+$Tregs unclear.<br>B cell related genes in PB and urine associated with kidney transplant tolerance in patients (112)
Ctsh	I/II	Skin, colon, lung and adipose tissue-Tregs (33) (mt)	Associated with mature $ST2^+$tissue-Treg (33)
Mef2c	I/II	Effector-Tregs in heart transplant tolerance (113) (mt)	A biological circuit of Mef2c/Mef2d /Hdac9 that controlls effector Treg suppressive function and maintaining allograft survival (113, 114).

Mrc1 (cd206)	I/II	Expressed in Spinal cord tissue with enhanced *Foxp3* in mice (115)	Function in Treg unclear.
Klrd1	I/II	Bcl11b deficient Tregs (116) (mt and ht)	Associated with programming of NK cell genes in Tregs (116)
Serpinb1a	I/II	Lung tumor-Tregs (68) (mt)	Function in Treg unclear.
Adam12	I	Naïve and effector- Tregs (117, 118) (mt)	Contribution on differentiation of naïve and effector Tregs (117, 118)
Cd36	I	Tumour-Tregs (61), CCR6^{+} effector/memory Tregs *(119)* (mt)	Mediated metabolic adaptation supporting Treg survival and function in tumours (61)
Tnfrsf13c *(Baff-r)*	I	Breast cancer specimen of patients with enhanced *Foxp3* expression (62)	Promotes Treg expansion (120)
Ccl6	III	Adipose-resident Treg (55) (mt)	Associated with Treg recruitment into tissues (55)
[¶¶¶]Mitoch-ondrial genes	III	Tregs with mitochondrial transcription factor A (Tfam) expression (121) (mt)	Associated with Treg maintenance in non-lymphoid tissue and in tumours (121, 122)
Slc35e4	III	CCR6^{+}effector/memory Tregs (119) (mt)	Function in Treg unclear
Spp1	III	brain-Tregs (123) (mt)	Repair of white matter after ischemic stroke (123)

[†]Up DEG: upregulated differentially expressed genes with false discovery rate (FDR) < 0.05. [‡]Treg subset: I: SP/CD127^{high}Treg; II: DLN/CD127^{high}Treg, III: graft-Treg. [§]I/II/III: including upregulated-DEG (FDR < 0.05) or with a tendency for enhanced gene expression in I/II/III Treg subsets compared to CD127$^{-/low}$Treg or naïve-Tregs. [¶]ht: human Treg. [#]mt: mouse Treg. **HLA-DR on effector Tregs: HLA-DR is a human MHC-II molecule. These effector Tregs were CD45RA^{-}CD4^{+}CD25^{high} from peripheral blood mononuclear cells (PBMCs) of healthy human donors. [††]: HLA-DR^{+}CD4^{+}CD25^{high}CD62L^{high}Tregs were from PBMCs. [‡‡]*Klrg1*: upregulated-DEG or tendency of enhanced gene expression of *Klrg1* on SP/CD127^{high}Treg, DLN/CD127^{high}Treg, and graft-Treg subsets compared to splenic naïve CD4^{+}T cells, naïve-Treg and CD127$^{-/low}$Treg subsets. [§§]AKI: acute kidney injury. [¶¶]Ig genes[a]*:* Immunoglobulin genes including *Iglv3* and *Jchain*. [##]Ig genes[b]: *Iglc1, Iglc2, Iglv1, lglv2 and Igkv6-32*. [¶¶¶]Mitochondrial genes: *mt-Nd1, mt-Nd2, mt-Nd4* and *mt-Cytb*.

Supplemental Table 3. The number of DEGs from 6 paired cross-comparisons

**Pairwise comparison**	**†DEGs all**	**DEGs upregulated**	**DEGs downregulated**
SP/CD127highTreg vs SP/Naïve-Treg	293	112	181
SP/CD127highTreg vs SP/CD127-Treg	456	217	239
DLN/CD127highTreg vs DLN/Naïve-Treg	296	218	78
DLN/CD127highTreg vs DLN/CD127-Treg	234	123	111
Graft-Treg vs SP/Naïve-Treg	649	125	524
SP/CD127highTreg vs Graft-Treg	820	564	256

†DEGs: differentially expressed genes (absolute log fold change > ±1.5 and *P* <0.01)

Supplemental Table 4. The dosages of adoptive-transferring Tregs and challenging $CD4^+$T cells into porcine-NICC transplant $Rag^{-/-}$ mice

Rag-/- Groups	No. of Rag-/-	Ratio (Treg/$CD4^+$T)	Dose of Tregs†	Dose of $CD4^+$T cells‡	No. of Rag-/- in each dose
Transplant only	11	N/A	N/A	N/A	N/A
$CD4^+$T cells	12	1:3	N/A	$6×10^4$<br>$2×10^5$<br>$2.3×10^5$<br>$3.3×10^5$<br>$3.4×10^5$<br>$4.5×10^5$<br>$5×10^5$<br>$6×10^5$	2<br>2<br>1<br>2<br>1<br>1<br>2<br>1
Naïve-Tregs	7	1: 3	$2.1×10^4$<br>$2×10^5$<br>$2.2×10^5$<br>$3.6×10^5$	$6.3×10^4$<br>$6×10^5$<br>$6.6×10^5$<br>$10.8×10^5$	2<br>3<br>1<br>1
Tolerant Tregs	7	1: 3	$1.1×10^5$<br>$1.5×10^5$<br>$2.2×10^5$<br>$2.3×10^5$	$3.3×10^5$<br>$4.5×10^5$<br>$6.6×10^5$<br>$6.9×10^5$	2<br>2<br>1<br>2
memory-like $CD127^{high}$Tregs	6	1: 3	$2.1×10^4$<br>$6.7×10^4$<br>$1.1×10^5$<br>$2.8×10^5$	$6.3×10^4$<br>2.1×105<br>$3.3×10^5$<br>$8.4×10^5$	1<br>1<br>2<br>2

†Naïve-Tregs ($CD4^+GFP/Foxp3^+$Tregs) were sorted from DEREG-mice without transplantation and no treatment; and tolerant-Tregs ($CD4^+GFP/Foxp3^+$Tregs) and memory-like $CD127^{high}$Tregs ($CD127^{high}CD44^+CD62L^-CD4^+GFP^+/Foxp3^+$Tregs) were sorted from transplanted DEREG-mice recipients that received CTLA4-Fc/MR-1 treatment day-100 post-transplant.

‡$CD4^+$T cells (nonFoxp3 $CD4^+GFP^-$T cells) were sorted from Ly5.1Foxp3GFP mice.

Supplemental Table 5. Antibodies for flow cytometric analysis, cell sorting and isolation

**Antibodies Flow Cytometric Analysis and Cell sorting**			
Antibody//Target	*Clone*	*Conjugate*	*Catalogue number, Supplier*
CD3	17A2	FITC	555274, BD Pharmingen
CD3	17A2	BUV737	564380, BD Bioscience
CD3e	145-2C11	eFluor450	48-0031-82, eBioscience
CD4	RM4-5	PerCP	561090, BD Bioscience
CD4	RM4-5	Pacific Blue	558107, BD Pharmingen
CD4	GK1.5	BUV395	565974, BD Bioscience
CD8a	53-6.7	PE	553032, BD Pharmingen
CD25	PC61	APC	557192, BD Bioscience
CD27	LG.3A10	APC	560691, BD Bioscience
CD39	24DMS1	PE	12-0391-82, eBioscience
CD44	IM7	BV421	563970, BD Bioscience
CD45	30-F11	BUV395	564279, BD Bioscience
CD45.1	A20	PE	110707, BioLegend
CD45.2	104	Alexa Fluor 700	109821, BioLegend
CD62L	MEL-14	PE	561918, BD Bioscience
CD69	H12F3	APC	553237, BD Pharmingen
CD103	M290	PE	557495, BD Bioscience
CD127	A7R34	APC-cy7	135040, BioLegend
H2kb	AF6-88.5	PE	553570, BD Pharmingen
STAT5	pY694	BV421	562984, BD Biosciences
IA/IE	M5/114.15.2	BV711	563414, BD Biosciences
Foxp3	FJK-16s	PE	12-5773-80B, eBioscience
CD16/CD32	2.4G2	Purified	553142, BD Biosciences
DAPI			2818-90-3, Sigma-Aldrich
Zombie Yellow			423104, BioLegend,
**CD4 T cell isolation**			
CD4	L3T4	Microbeads	130-117-043, Miltenyi Biotec,

Supplemental Table 6: Immunohistochemistry and immunofluorescence staining antibodies

**Primary Antibodies**		
Antibody//Target	*Concentration*	*Catalogue number, Supplier*
Guinea pig Anti-Insulin (polyclonal)	Ready-to-Use	IR00261-2, Agilent Dako
Rat Anti-Mouse CD16/CD32 (2.4G2, Fc Block)	5µg/mL	553142, BD Biosciences
**Secondary Antibodies and Counterstain**		
Rabbit Anti-Guinea pig immunoglobulins/HRP	6.5µg/mL	P0141, Agilent Dako
Goat Anti-Guinea pig Texas Red	10µg/mL	Ab6906, abcam
DAPI (Vectashield Mounting Medium)	1.5µg/mL	H-1200-10, Vector Laboratories

Supplemental Table 7. Real-time RT polymerase chain reaction (RT-PCR).

Gene name	*Assay ID*	*Supplier*
*Il1**2***	Mm00434256_m1	Thermo Fisher Scientific
Il7	Mm01295803_m1	Thermo Fisher Scientific
Il10	Mm00439614_m1	Thermo Fisher Scientific
Il33	Mm00505403_m1	Thermo Fisher Scientific
Tgfb1	Mm00441724_m1	Thermo Fisher Scientific
Ifng	Mm99999071_m1	Thermo Fisher Scientific
Blimp1(*Prmd1)*	Mm00476128_m1	Thermo Fisher Scientific
Ctla4	Mm01253995_m1	Thermo Fisher Scientific
Ebi3	Mm00469294_m1	Thermo Fisher Scientific
Havcr2 (*Tim3*)	Mm01294183_m1	Thermo Fisher Scientific
Hprt	Mm03024075_m1	Thermo Fisher Scientific
Gapdh	4352932E-0808024)	Applied Biosystems Thermo Fisher Scientific

Supplemental Table 8. Imaging mass cytometry antibodies

Antibody/Target	*Conjugate*	*Concentration*	*Catalogue number, Supplier*
CD45 (30-F11)	113In	4µg/mL	553076, BD Biosciences
CD69 (H1.2F3)	139La	8µg/mL	104502, Biolegend
CD11c (N418)	142Nd	2µg/mL	117302, Biolegend
CD4 (RM4-5)	145Nd	4µg/mL	100506, Biolegend
F4/80 (BM8)	146Nd	8µg/mL	123102, Biolegend
IA/IE (M5/114.15.2)	150Nd	2µg/mL	107602, Biolegend
CD3e (145-2C11)	152Sm	4µg/mL	100302, Biolegend
CD62L (Polyclonal)	160Gd	2µg/mL	PA595721, ThermoFisher
CD25 (3C7)	161Dy	8µg/mL	101902, Biolegend
FOXP3 (FJK-16s)	164Dy	4µg/mL	14-5773, eBioscience (ThermoFisher)
CD27 (LG.3A10)	167Er	4µg/mL	124202, Biolegend
CD8a (53-6.7)	168Er	4µg/mL	100755, Biolegend
CD44 (IM7)	171Yb	0.5µg/mL	553131, BD Biosciences
CD127 (A7R34)	175Lu	4µg/mL	135002, Biolegend
CD45R/B220(RA3-6B2)	176Yb	2µg/mL	103202, Biolegend
DNA	191Ir and 193Ir	0.625µM	201192A, Fluidigm

Supplemental Table 9. Software for imaging mass cytometry analysis

Software	*Author*	*Source*
MCD Viewer v1.0.560.2	Fluidigm	https://www.fluidigm.com/software
CellProfiler v3.1.9	Broad Institute	https://cellprofiler.org/
Ilastik v1.3.3	Heidelberg University	https://www.ilastik.org/
Anaconda Navigator v1.9.12	Anaconda	https://www.anaconda.com/products/individual
IMC Segmentation Pipeline v2.0	Bodenmiller lab	https://github.com/BodenmillerGroup/ImcSegmentationPipeline
CellProfiler IMC Plugins	Bodenmiller lab	https://github.com/BodenmillerGroup/ImcPluginsCP
Modified Bodenmiller Segmentation Pipeline	Ashurst, T; Sydney Cytometry Facility	https://sydneycytometry.org.au/wiki-launch
histoCAT v1.76	Bodenmiller lab	https://bodenmillergroup.github.io/histoCAT/
R v4.0.3	R Core Team	https://cran.csiro.au/
RStudio v1.4.1103	RStudio	https://rstudio.com/
Spectre v0.3.7	Sydney Cytometry Facility	https://github.com/sydneycytometry/Spectre
ImageJ v1.52a	NIH	https://imagej.nih.gov/ij/download.html

References

95. Ashhurst TM, et al. Integration, exploration, and analysis of high-dimensional single-cell cytometry data using Spectre. *Cytometry A.* 2021.
96. Zhang R, et al. Tissue Treg Secretomes and Transcription Factors Shared With Stem Cells Contribute to a Treg Niche to Maintain Treg-Ness With 80% Innate Immune Pathways, and Functions of Immunosuppression and Tissue Repair. *Front Immunol.* 2020;11:632239.
97. Layland LE, et al. Pronounced phenotype in activated regulatory T cells during a chronic helminth infection. *J Immunol.* 2010;184(2):713-24.
98. Zhang N, et al. Regulatory T cells sequentially migrate from inflamed tissues to draining lymph nodes to suppress the alloimmune response. *Immunity.* 2009;30(3):458-69.
99. Cuadrado E, et al. Proteomic Analyses of Human Regulatory T Cells Reveal Adaptations in Signaling Pathways that Protect Cellular Identity. *Immunity.* 2018;48(5):1046-59 e6.
100. Wienke J, et al. Human Tregs at the materno-fetal interface show site-specific adaptation reminiscent of tumor Tregs. *JCI Insight.* 2020;5(18).
101. Neumann C, et al. c-Maf-dependent T(reg) cell control of intestinal T(H)17 cells and IgA establishes host-microbiota homeostasis. *Nat Immunol.* 2019;20(4):471-81.
102. Yuan X, et al. The importance of regulatory T-cell heterogeneity in maintaining self-tolerance. *Immunol Rev.* 2014;259(1):103-14.
103. Shalev I, et al. Targeted deletion of fgl2 leads to impaired regulatory T cell activity and development of autoimmune glomerulonephritis. *J Immunol.* 2008;180(1):249-60.
104. Konieczny BT, et al. IFN-gamma is critical for long-term allograft survival induced by blocking the CD28 and CD40 ligand T cell costimulation pathways. *J Immunol.* 1998;160(5):2059-64.
105. Wood KJ, and Sawitzki B. Interferon γ: a crucial role in the function of induced regulatory T cells in vivo. *Trends in Immunology.* 2006;27(4):183-7.
106. Chu M, et al. Nebulin, a multi-functional giant. *J Exp Biol.* 2016;219(Pt 2):146-52.
107. Agenès F, et al. Differential expression of regulator of G-protein signalling transcripts and in vivo migration of CD4+ naïve and regulatory T cells. *Immunology.* 2005;115(2):179-88.
108. Guo X, et al. Global characterization of T cells in non-small-cell lung cancer by single-cell sequencing. *Nat Med.* 2018;24(7):978-85.
109. Giordano G, et al. JAK/Stat5-mediated subtype-specific lymphocyte antigen 6 complex, locus G6D (LY6G6D) expression drives mismatch repair proficient colorectal cancer. *J Exp Clin Cancer Res.* 2019;38(1):28.
110. Semba T, et al. Lung Adenocarcinoma Mouse Models Based on Orthotopic Transplantation of Syngeneic Tumor-Initiating Cells Expressing EpCAM, SCA-1, and Ly6d. *Cancers (Basel).* 2020;12(12).
111. Gerner MC, et al. The TGF-b/SOX4 axis and ROS-driven autophagy co-mediate CD39 expression in regulatory T-cells. *FASEB J.* 2020;34(6):8367-84.
112. Newell KA, et al. Identification of a B cell signature associated with renal transplant tolerance in humans. *The Journal of clinical investigation.* 2010;120(6):1836-47.
113. Di Giorgio E, et al. A Biological Circuit Involving Mef2c, Mef2d, and Hdac9 Controls the Immunosuppressive Functions of CD4+Foxp3+ T-Regulatory Cells. *Front Immunol.* 2021;12:703632.
114. Di Giorgio E, et al. MEF2D sustains activation of effector Foxp3+ Tregs during transplant survival and anticancer immunity. *The Journal of clinical investigation.* 2020;130(12):6242-60.
115. Fischer R, et al. TNFR2 promotes Treg-mediated recovery from neuropathic pain across sexes. *Proc Natl Acad Sci U S A.* 2019;116(34):17045-50.
116. Drashansky TT, et al. Bcl11b prevents fatal autoimmunity by promoting T(reg) cell program and constraining innate lineages in T(reg) cells. *Sci Adv.* 2019;5(8):eaaw0480.

117. Liu Y, et al. A critical function for TGF-beta signaling in the development of natural CD4+CD25+Foxp3+ regulatory T cells. *Nat Immunol.* 2008;9(6):632-40.
118. Zhou AX, et al. The metalloprotease ADAM12 regulates the effector function of human Th17 cells. *PLoS One.* 2013;8(11):e81146.
119. Zhao J, et al. MicroRNAs expression profile in CCR6(+) regulatory T cells. *PeerJ.* 2014;2:e575.
120. Stohl W, and Yu N. Promotion of T Regulatory Cells in Mice by B Cells and BAFF. *J Immunol.* 2020;204(9):2416-28.
121. Fu Z, et al. Requirement of Mitochondrial Transcription Factor A in Tissue-Resident Regulatory T Cell Maintenance and Function. *Cell Rep.* 2019;28(1):159-71 e4.
122. Weinberg SE, et al. Mitochondrial complex III is essential for suppressive function of regulatory T cells. *Nature.* 2019;565(7740):495-9.
123. Shi L, et al. Treg cell-derived osteopontin promotes microglia-mediated white matter repair after ischemic stroke. *Immunity.* 2021;54(7):1527-42 e8.
